# Self-Aligned Crystallographic Multiplication of Nanoscale Silicon Wedges for High-Density Fabrication of 3D Nanodevices

**DOI:** 10.1021/acsanm.2c04079

**Published:** 2022-10-12

**Authors:** Erwin Berenschot, Roald M. Tiggelaar, Bjorn Borgelink, Chris van Kampen, Cristian S. Deenen, Yasser Pordeli, Haye Witteveen, Han J. G. E. Gardeniers, Niels R. Tas

**Affiliations:** † Mesoscale Chemical Systems, MESA+ Institute, University of Twente, Drienerlolaan 5, 7522 NB Enschede, The Netherlands; ‡ NanoLab Cleanroom, MESA+ Institute, University of Twente, Drienerlolaan 5, 7522 NB Enschede, The Netherlands

**Keywords:** 3D nanofabrication, self-aligned fabrication, crystallographic nanolithography, corner lithography, edge lithography, silicon crystal, silicon wedges

## Abstract

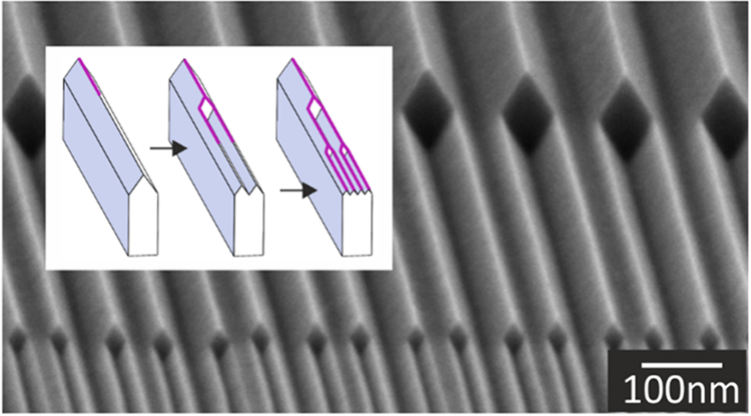

High-density arrays of silicon wedges bound by {111} planes on silicon (100) wafers have been created by combining convex corner lithography on a silicon dioxide hard mask with anisotropic, crystallographic etching in a repetitive, self-aligned multiplication procedure. A mean pitch of around 30 nm has been achieved, based on an initial pitch of ∼120 nm obtained through displacement Talbot lithography. The typical resolution of the convex corner lithography was reduced to the sub-10 nm range by employing an 8 nm silicon dioxide mask layer (measured on the {111} planes). Nanogaps of 6 nm and freestanding silicon dioxide flaps as thin as 1–2 nm can be obtained when etching the silicon at the exposed apices of the wedges. To enable the repetitive procedure, it was necessary to protect the concave corners between the wedges through “concave” corner lithography. The produced high-density arrays of wedges offer a promising template for the fabrication of large arrays of nanodevices in various domains with relevant details in the sub-10 nm range.

## Introduction

Self-aligned patterning techniques such as edge lithography (EL)^1,2^ have become indispensable to reach smaller device footprints in state-of-the-art electronic nanosystem fabrication.^3^ In self-aligned dual patterning,^4^ masking features are produced at the sidewalls of initial photoresist line patterns, thereby doubling the number of lines. This procedure can be repeated for quadruple patterning.^4^ Important recent innovations include EL based on exposure of overlapping positive and negative photoresists to create subwavelength edge features in so-called dual-layer photolithography,^5^ SADP-based active trim contact formation with anisotropic pattern pitches,^6^ and crossed self-aligned multipatterning strategies for staggered hole array creation,^7^ both typically for DRAM production.

In recent years, a related self-aligned nanopatterning technique has been introduced based on silicon nitride left in sharp concave corners after conformal deposition and isotropic thinning.^8−10^ This so-called corner lithography (CL) is functional in concave corners independent of their orientation in space and can be repetitively applied. CL allows the formation of rather complicated 3D nanostructures in a self-multiplying process when combined with anisotropic etching of a single crystalline silicon mold.^11^ While shaping the underlying silicon is often the aim of a self-aligned patterning step, we found that use of the crystallographic properties of the silicon substrate can be key to enhance the quality of this step.^12^ Extremely sharp corners and smooth sidewalls can be formed through anisotropic etching that ends on slow-etching crystal planes, thereby enabling the formation of near ideal mold structures for self-aligned techniques such as EL and CL.^8,10,13,14^ This fabrication strategy is referred to as “crystallographic nanolithography.” Typically, anisotropic etching is used to sharply define the geometry confined by the smooth slow-etching crystal planes of known orientation. The self-aligned nanopatterning techniques have the function to create nanoscale masks in 3D for the subsequent anisotropic etching step. This directs the formation of more complex 3D nanostructures.

Initially, CL was applied in sharp concave corners. Recently, a new self-aligned patterning technique, viz. “convex CL,” was introduced.^15,16^ It is based on the principle that slightly thinner silicon dioxide (SiO_2_) layers are formed near and on the apex of sharp convex corners during low-temperature thermal oxidation (typically <900 °C).^17,18^ After isotropic thinning of this SiO_2_ in hydrofluoric acid, the convex silicon corners can selectively be exposed and subsequently machined. By selectively exposing the sharp convex corners of oxidized silicon wedges, arrays of sub-20 nm nanogaps were created.^15,16^ In the current work, we further explore and develop the combination of convex CL with silicon wedges toward an advanced substrate for self-aligned device fabrication.

Literature about silicon wedges goes back to the late 80s. Gray et al.^19^ and Campisi et al.^20^ used orientation-dependent etching (ODE) of crystalline silicon (Si) to fabricate point- and wedge-like field emitter arrays for vacuum field effect transistors. Arrays of wedge-like field emitters were made in (100)-oriented silicon using potassium hydroxide (KOH) as an etchant in combination with a mask pattern of stoichiometric silicon nitride. The resulting wedges were defined by slow-etching {111} planes. Upon applying an oxidation-sharpening step, the radius of curvature of the convex corner of 10–50 μm long wedges could be reduced to <15 nm.^21,22^ Hashiguchi et al. appointed two limitations of this ODE process, i.e., its large complexity and the use of time-stop under-etching for shaping of the convex structures, and proposed an ODE process in combination with local oxidation of silicon (LOCOS) to overcome these shortcomings.^23,24^ With this LOCOS-based process, wedges were realized as follows: a rectangular pattern in silicon nitride (SiN), with one edge aligned parallel to the ⟨110⟩ direction of the (100) Si substrate, was created with reactive ion etching (RIE) and used as a mask for selective etching in KOH. Subsequently, the etched pattern was thermally oxidized, followed by removal of SiN, and a second anisotropic etching step. This LOCOS-based process yielded a sharp convex corner with a radius of curvature of ca. 3 nm for 0.3 mm long wedges, since the edges, located below the surface of the substrate, were formed by two intersecting {111} planes and sharpened by oxidation.^24^ The versatility of this process is apparent from the manufacturing of various structures, such as quantum wires,^25^ conical field emitters,^26^ and wafer-scale nanowires.^27^ Over the years, Hashigushi’s LOCOS-based wedge fabrication method^23,24^ was modified/optimized by multiple groups. The main adjustment was the prevention of flat intergroove spacers between wedges,^28−30^ resulting in a higher areal density. For the two anisotropic etch steps, KOH and tetramethylammonium hydroxide (TMAH) were used as etchants, which both yield {111} Si-terminated structures. This method, which is based on controllable etching in the ⟨111⟩ direction,^6,31^ is used to fabricate a variety of structures with microscale to nanoscale dimensions. For example, Ribbing et al. employed it to achieve saw-tooth refractive X-ray lenses,^28^ Jin et al. to create nanoslit arrays for surface-enhanced Raman spectroscopy with size-controllable gaps at the apex upon coverage with gold (Au),^29^ and Wilbers et al. to manufacture high-density ordered arrays of crystalline silicon nanostructures.^30^

Here, we extend the application of crystallographic nanolithography on the sharp convex corners of silicon wedges to the sub-10 nm scale. Furthermore, we introduce a novel self-aligned multiplication scheme which results in increasing the density of silicon wedges by a factor 4 to an average 30 nm pitch. Nanogaps down to 6.5 nm are produced by this technique, based on an initial silicon dioxide thickness of 7.9 nm on the {111} planes of the wedges. It is shown that after thinning of the oxide and etching of the exposed convex silicon corner, freestanding oxide flaps with a tunable length and as thin as 2 nm can be realized. We envision that the reported results will enable the self-aligned batch fabrication of a variety of devices, e.g., in the nanofluidic, nanomechanical, nanoelectronic, and nanophotonic domains.

## Experimental Section and Results and Discussion

**Figure 1:**
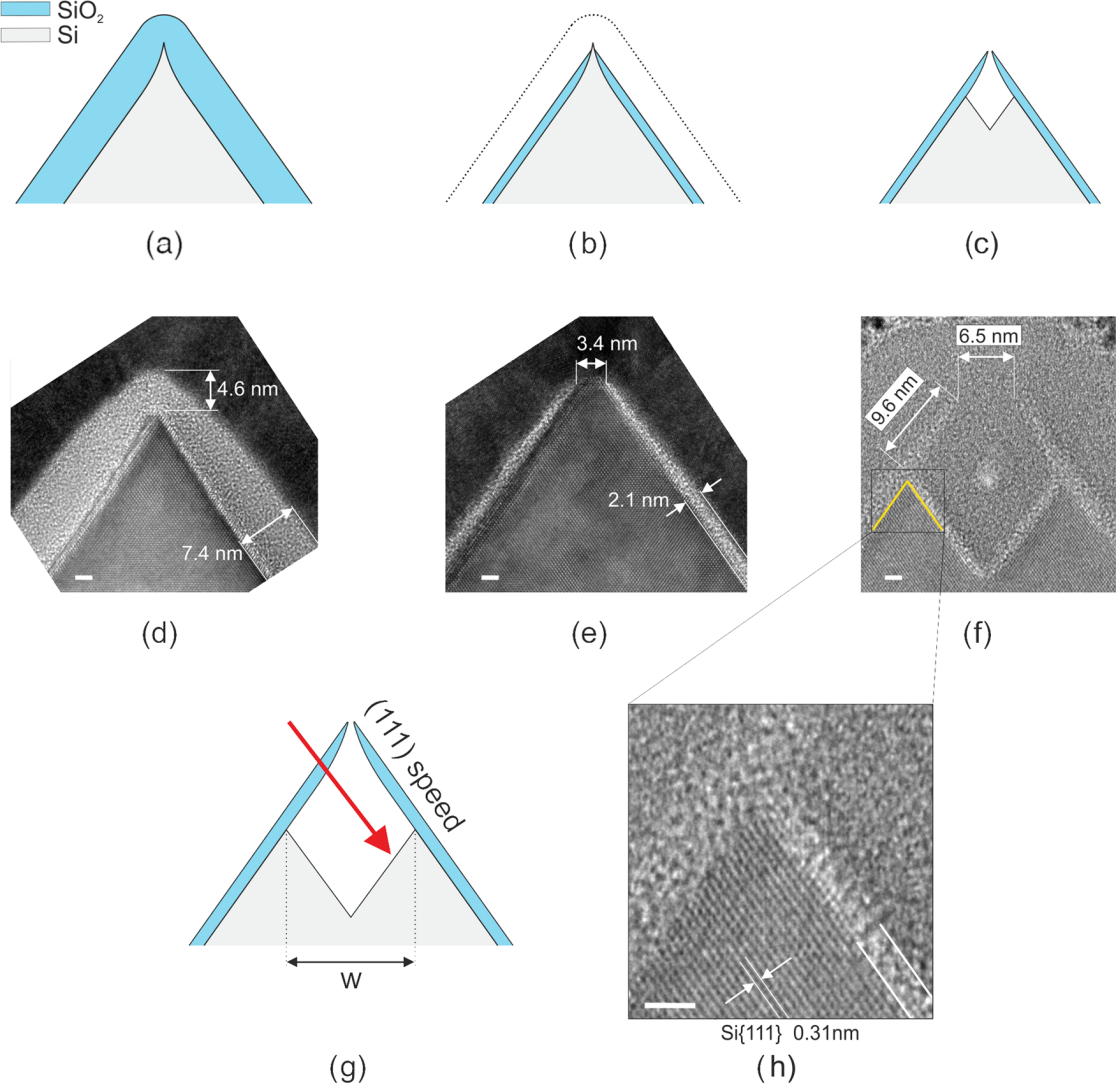
Principle of convex corner lithography and cavity etching applied to silicon wedges (scale bar TEM images: 2 nm), with (a) low-temperature thermal oxidation; (b) isotropic thinning of silicon oxide; (c) anisotropic etching of silicon from the exposed apex; (d, e) TEM images belonging to steps (a) and (b), respectively; (f) TEM image after step (c) and after embedding the freestanding silicon oxide flaps in LPCVD silicon nitride; (g) illustrating the direction of the ⟨111⟩ etching speed; and (h) zoom-in near the apex of a newly formed next-generation wedge after wet chemical cleaning and subsequent embedding in LPCVD silicon nitride.

The principle of the basic wedge machining method is illustrated in Figure 1, based on initial experiments with a SiO_2_ mask thickness of 30 nm.^15^ In the current work, wedges bound by {111} Si planes were created as described in ref (30) (the general process for nanowedge fabrication is given in Figure S1). Next, they were steam oxidized at 800 °C for 15 min to create ca. 8 nm thick SiO_2_ on the exposed {111} planes (Figure 1a,d). The oxide was isotropically etched in a 1% hydrofluoric acid (HF) solution for 67 s until a layer of ca. 2 nm SiO_2_ remained on the {111} planes (Figure 1b,e). This etch step exposed the silicon apices of the wedges, which were then selectively etched in TMAH (25 wt %, 70 °C). After “inversion” of the apices, TMAH slowly etches into the ⟨111⟩ directions of silicon, yielding a cavity with a diamond-shaped cross-sectional appearance (Figure 1c,f). Due to the finite (i.e., non-zero) etching rate of SiO_2_ in TMAH, during the TMAH etch step, the thickness of the ca. 2 nm thick SiO_2_ layer was reduced to 1.3 ± 0.3 nm, but such thickness appeared to be rigid enough to create stable freestanding flaps. The dimensional enlargement of this cavity as a function of the etch time in TMAH has been characterized by analyzing three cross-sections with scanning electron microscopy (SEM), and the results (Table 1) indicate a ⟨111⟩ etch rate (Figure 1g) of about 10 nm/min. For transmission electron microscopy (TEM) analysis, the etching was stopped after 1 min and the resulting structure was encapsulated in 13 nm of low-pressure chemical vapor deposited (LPCVD) silicon nitride (Si_3_N_4_) to provide mechanical support to the thin freestanding SiO_2_ flaps during the TEM sample preparation. A cross-sectional slice was formed by focused ion beam (FIB) etching and subsequently imaged (Figure 1f,h). The TEM image confirms that freestanding oxide flaps of about 2 nm in thickness and 10 nm in length had been formed with a gap of 6–7 nm. It also shows that the initial wedge is split into two wedges, each being near atomically sharp at the apex, which suggested and inspired us to explore repetition of this wedge nanomachining procedure. Additional TEM images and atomic force microscopy (AFM) data obtained at various stages of the wedge nanomachining procedure are shown in Figure S2.

**Table 1:** Etch Rate Si ⟨111⟩ in 25% TMAH (70 °C)

etch time [s]	width *w* [nm]	expansion rate of the width (*w*) of the parallelepiped cavity [nm/min]	Si ⟨111⟩ etch rate [nm/min]
120	25 ± 2	12.5	10.8
240	45 ± 2	11.3	9.7
330	64 ± 2	11.6	10.0

As can be seen in Figure 1, the grown silicon dioxide layer thickness in the proximity of silicon corners appears to be thinner than on flat silicon planes.^18^ This retardation effect is explained using existing models, which were developed to describe the silicon dioxide layer thickness on crystalline silicon cylinders with feature sizes down to 1 μm^17,32^ and with an outer and inner radius down to 240 and 70 nm,^33^ respectively. At convex silicon corners, the diffusion flux of oxidant species reaching the silicon is larger than at flat silicon surfaces or concave silicon corners, due to the larger opening angle. Moreover, mechanical stress accumulating in the silicon dioxide layer alters the growth dynamics. Normal to the silicon interface, a stress exists in the silicon dioxide layer of which the sign (i.e., compressive or tensile) depends on the type of corner.^32^ The lateral stress depends on the radius of curvature of the silicon corner and is tensile at convex silicon corners and compressive at concave corners.^32^ Because the grown SiO_2_ layer is pushed outward for convex corners and inward for concave corners, the result is either a reduction of the lateral stress for convex corners or a reversion for concave corners. The pressure inside the grown layer is proportional to the sum of the normal and tangential stress^18,32−34^ (see Figure S3 for the definition of directions) and affects the reaction rate of silicon with oxidant species. For thin silicon dioxide layers as utilized in this work (i.e., sub-10 nm scale thicknesses), the tangential stress is dominant because the normal stress becomes small for these thickness values.^32^ This tangential stress is not identical for convex and concave corners, i.e., the tangential stress in a convex corner exceeds the value for a concave corner,^33^ which affects the thickness of the grown layer.^33^ In the presented work, the silicon dioxide layers are wet (i.e., in steam atmosphere) thermally grown at 800 °C. At these processing conditions, stress relaxation by viscous flow is assumed to be negligible.^35^ It is emphasized that the growth models are based on circular objects, i.e., cylinders, and have not been developed for corners. Moreover, at this sub-10 nm scale, other mechanisms might become more prominent in determining the growth process, such as reordering of the silicon atoms at the interface due to the large lateral stress.^36^

**Figure 2:**
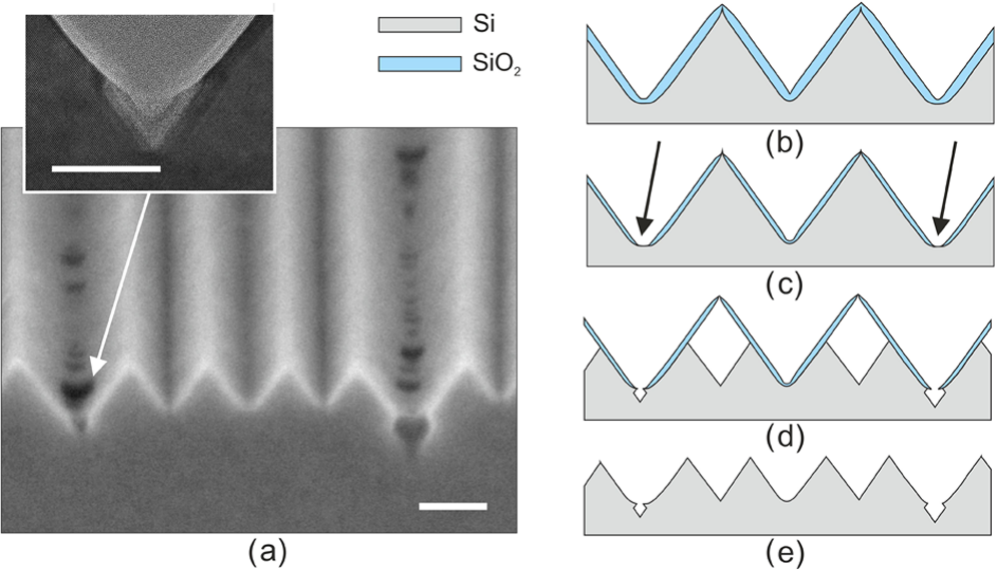
Repetitive wedge nanomachining procedure without modifications leads to an improper SiO_2_ mask function in concave corners, due to which etching defects are formed during TMAH etching, which in (a) can be seen in the first and the fifth grooves, counting from the left (scale bar: 100 nm). The steps leading to this issue as shown in panel (a) are shown in (b–e), with (b) low-temperature thermal oxidation, (c) HF thinning of the silicon oxide, (d) TMAH etching of the exposed silicon and parasitic thinning and breakthrough of the silicon oxide in concave corners, and (e) after stripping the silicon oxide.

Initially, the repetitive wedge machining procedure was implemented in a straightforward manner, meaning that after formation of the second-generation wedges the silicon dioxide hard mask was removed with HF. However, periodicity doubling of the wedges by convex corner lithography and the subsequent anisotropic etching of the exposed silicon resulted in a high defect density close to the concave silicon corners (Figure 2). After the hard mask was removed in HF, the concave silicon corner in the first wedge generation appeared to be rounded off. This phenomenon was expected because silicon is consumed during the growth of the silicon dioxide layer, resulting in an increased radius of curvature of the concave corner. Due to the increase of the radius of curvature, the concave corner consists of slower oxidizing {100}-like crystal planes. After the growth of the second silicon dioxide hard mask, its thickness at these concave corners is approximately equal to its thickness at the convex silicon corners. Hence, after employing convex corner lithography, the silicon dioxide layer at the concave corners becomes too thin (i.e., ≤0.6 nm; based on Figures 2 and S2b1) to act successfully as a hard etching mask, for which reason the wedge machining procedure has to be modified for successful use in a repetitive manner.

**Figure 3:**
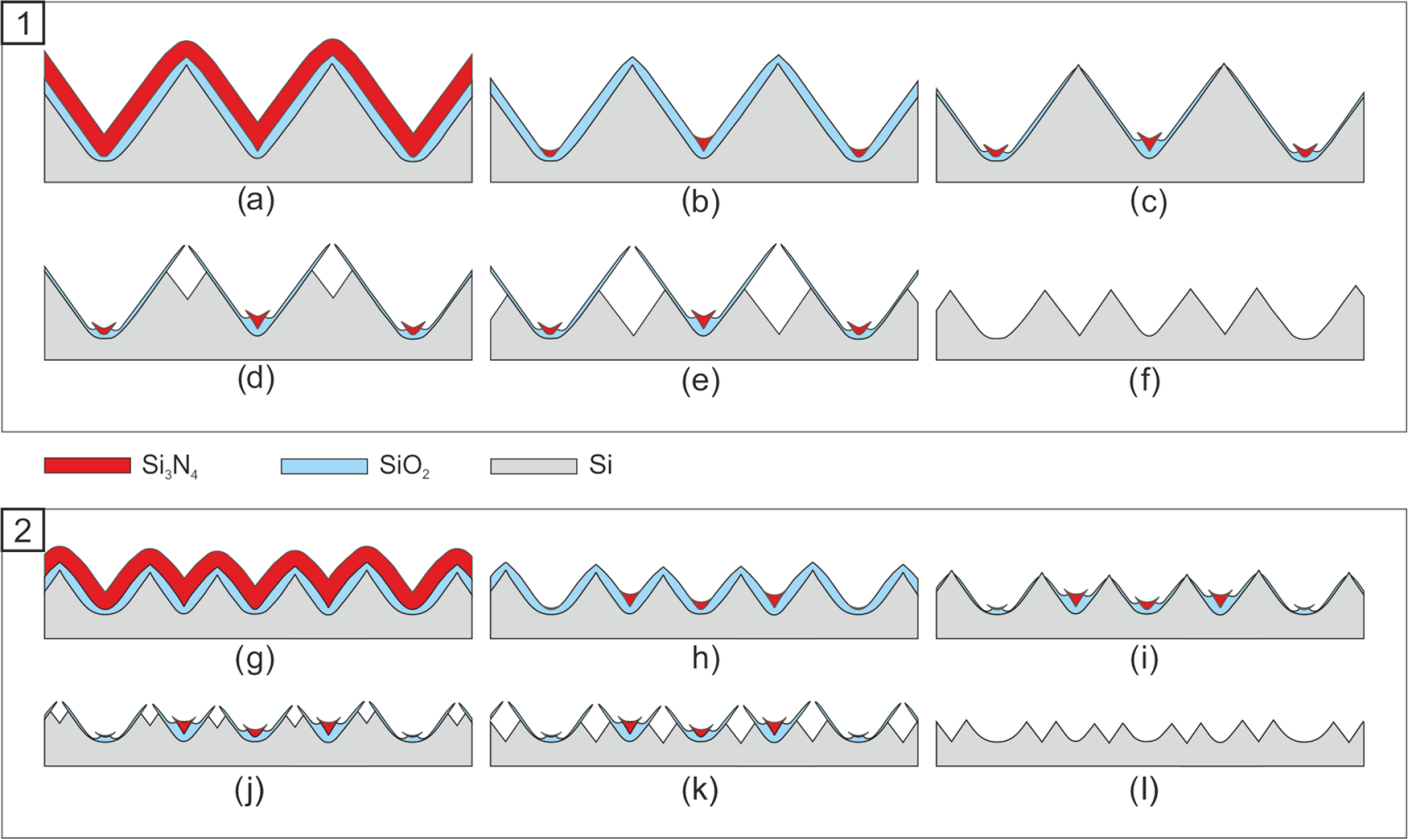
Nanomachining procedure for self-aligned multiplication of silicon wedges by crystallographic nanolithography. The detailed steps are (a) low-temperature thermal oxidation and LPCVD silicon nitride on the initial wedge structures, (b) concave corner lithography through isotropic thinning of the silicon nitride, (c) convex corner lithography by isotropic thinning of the silicon oxide, (d, e) secondary wedge formation by TMAH etching, (f) stripping of the hard mask layers, and (g–l) repetition of panels (a–f) for tertiary wedge formation.

To mitigate this problem, a concave corner lithography step^2−4^ is introduced after the formation of the (low temperature) silicon dioxide mask (Figure 3a,b) and before applying the convex corner exposure etching in HF (Figure 3c) and cavity etching in TMAH (Figure 3d,e). The added corner lithography step utilizes stoichiometric silicon nitride (LPCVD), which is isotropically etched in a hot phosphoric acid (H_3_PO_4_) solution. The temperature (140 °C) and concentration (85 wt %) of this solution were optimized for selectivity in etching Si_3_N_4_ with respect to SiO_2_. Both these masking materials can be completely removed in 50 vol % HF (Figure 3f), after which the complete procedure can be repeated (Figure 3g–l). Upon performing this sequence twice, the initial wedge pitch of ∼120 nm can be reduced to an average of 30 nm. It is noted that a 3D representation of this double-repeated self-aligned multiplying wedge nanomachining procedure is shown in Figure S4.

**Figure 4:**
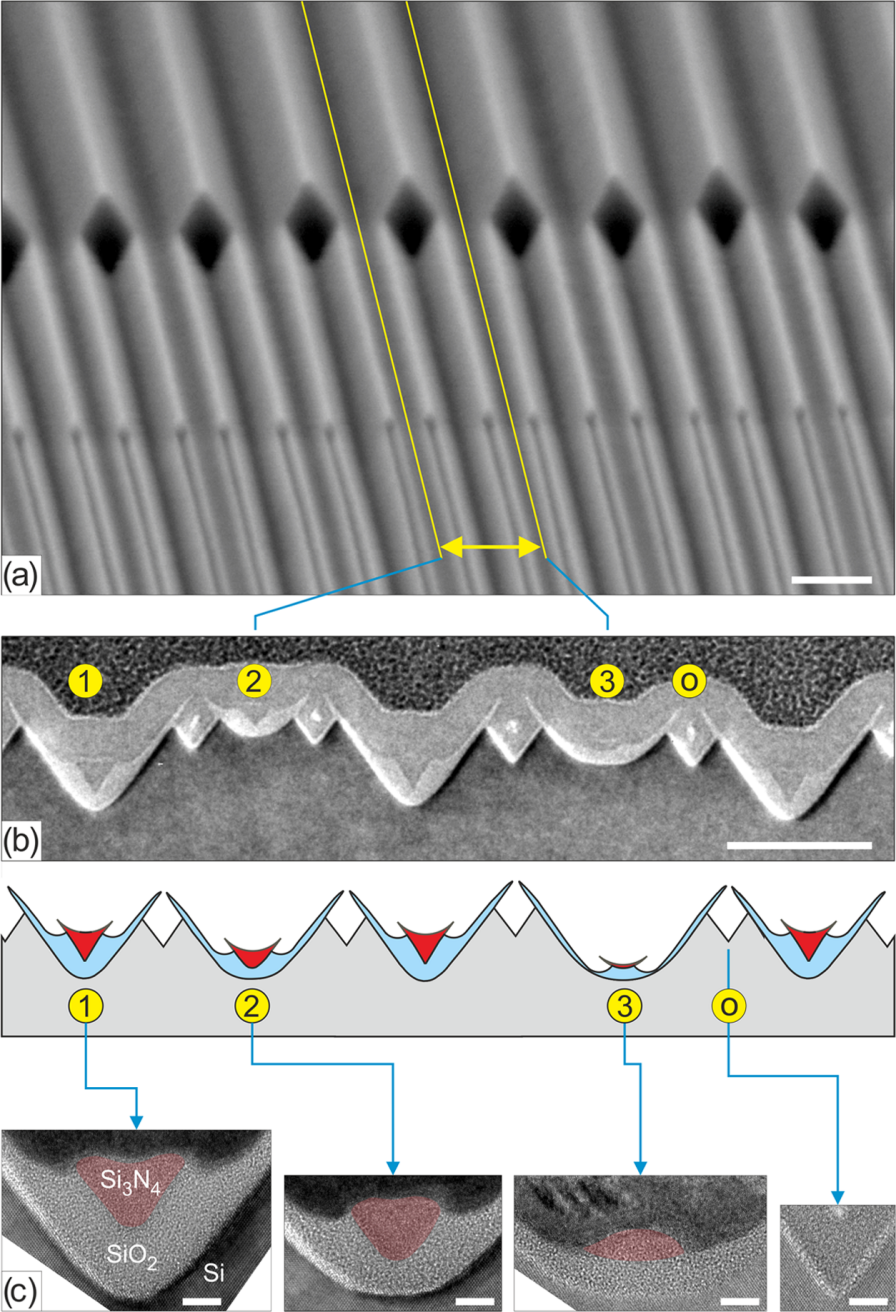
SEM and TEM images of triple-repeated self-aligned multiplying wedge nanomachining procedure (scale bars: (a) SEM 100 nm, (b) TEM 50 nm, and (c) TEM 5 nm). The numbers indicate the number of times a concave corner was exposed to the thermal oxidation–HF stripping sequence.

To verify the outcome of this repetitive nanomachining procedure, UV-lithography masking (with line dimensions in the micrometer range) was employed in steps 3b and 3h (see Figure 3), such that only in selected areas the silicon dioxide is etched and doubling of the wedges could occur. The resulting structure is shown in the SEM image in Figure 4a, illustrating the different “generations” of wedges produced. To analyze the structure in more detail, a cross-sectional slice was made by FIB etching and imaged with TEM (Figure 4b), after embedding the structure in a LPCVD silicon nitride support matrix. A close look at this TEM image reveals that apices of the wedges appear sharp, while the concave “bottom” corners have different radii of curvature. The observed differences in radii of curvature correlate with the number of times the various concave corners were oxidized followed by removal of the formed SiO_2_ in an HF-based etchant. These numbers are given in Figure 4b,c (zoom-in TEM images) at the various corners (after the final TMAH etching step): the higher the number, the larger the radius of curvature of the (initially sharp) concave corner (because of the increased amount of times that the sequence “oxidation and HF etching” was performed). It has to be noted that the uniformity of this wafer-scale wedge nanomachining procedure is very high, as evidenced by analysis of top-view SEM images and TEM images (Section 5 of the Supporting Information). In a separate, repeated run, the pitch of wedges after doubling was determined to be 31.0 ± 1.1 nm (1 SD) where 25 locations distributed over the whole four inch wafer were considered. This gives an indication for the combined uniformity of the convex corner lithography with the subsequent anisotropic etching step.

**Figure 5:**
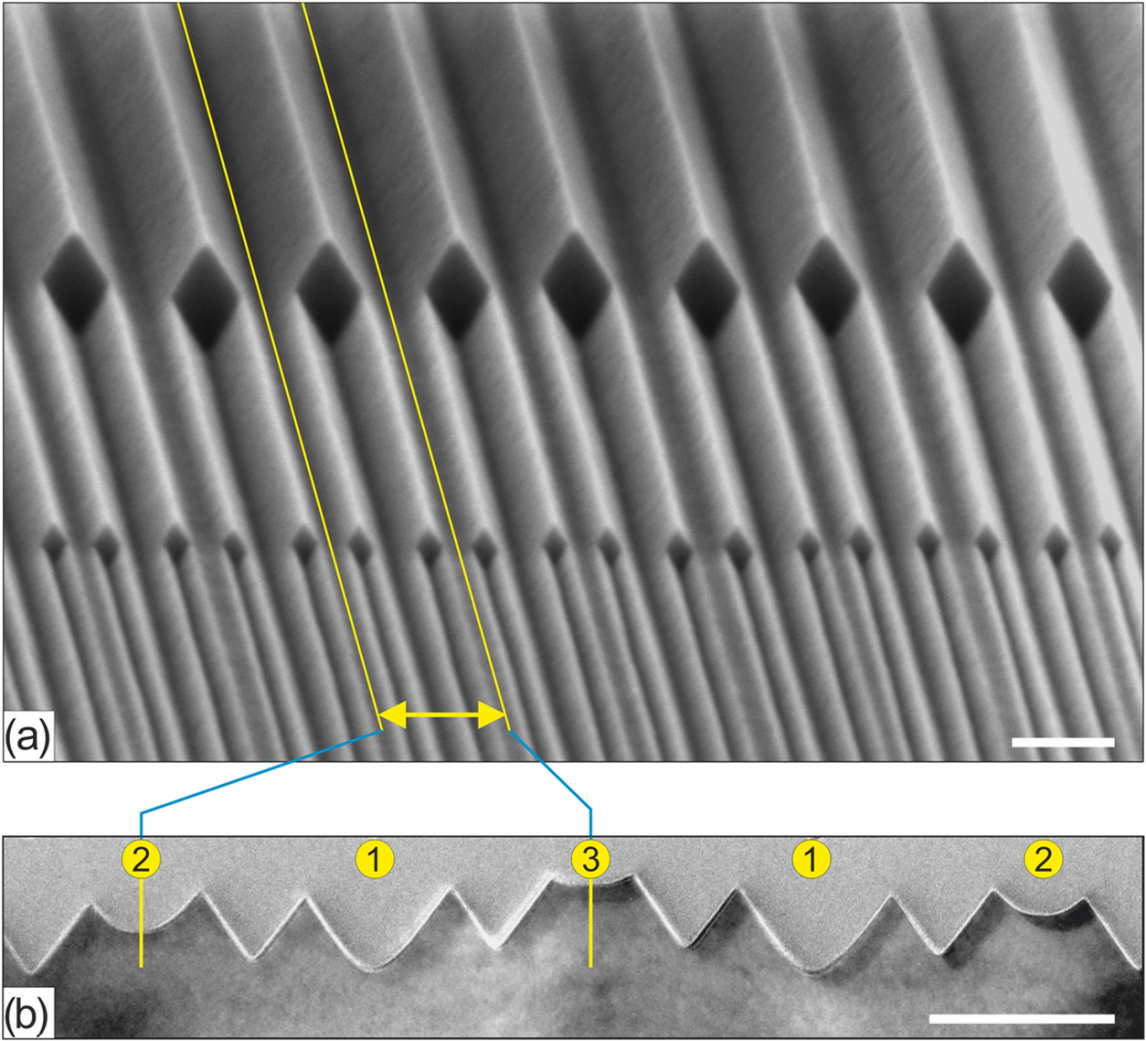
SEM and TEM images of triple-repeated self-aligned multiplying wedge nanomachining procedure with tuned 50% duty-cycle (scale bar: (a) 100 nm and (b) 50 nm). Again, the numbers indicate the number of times that a concave corner was exposed to the thermal oxidation–HF stripping sequence.

Two other aspects have to be addressed as well upon inspecting Figure 4. The first aspect concerns the depths and the pitch of the different generations of cavities etched at the apices of wedges. The width of the grooves is nonuniform (Figure 4b); however, by tuning the timespan of the steps during which these cavities are created (Figure 3d,e,j,k), it is possible to realize wedge generations with (nearly) uniform pitches. In case of Figure 4b, the etch times of the second and third anisotropic silicon etch steps were extended to achieve this. Figure 5 shows images of triple-repeated self-aligned multiplying wedges with a close to 50% duty-cycle. It is noted that, as abovementioned, the number of times that a concave corner is oxidized (followed by removal of the oxide) affects the radius of curvature of a concave corner (Figure 4c); as a consequence, it is difficult to achieve identical depths for all generations of cavities in case of 50% duty-cycle self-aligned multiplying wedges (Figure 5b). The silicon dioxide layer thickness can be reduced as long as the grown silicon dioxide is 2 nm thicker at the slanted {111} Si planes with respect to its thickness at the convex corners. In this work, steam oxidation of silicon at 800 °C yielded a silicon dioxide layer thickness of 7.9 ± 0.4 and 5.7 ± 0.3 nm on (111) Si and (100) Si monitor substrates, respectively (see Section 2 (Supporting Information) and Figure S2). Deduced from the data presented by Massoud et al.,^37,38^ a layer thickness of ca. 6.2 nm ((111) Si) and ca. 4.0 nm ((100) Si) can be obtained if dry (i.e., in an oxygen–nitrogen mixture) thermal oxidation at 800 °C is used. This shows that the SiO_2_ thickness ratio between {111} Si vs {100} Si is increased if dry oxidation is used, meaning that the resulting radius of curvature at the concave corners is lower after wedge multiplication.

The second aspect concerns the so-called bird’s beak phenomenon,^39^ which occurs upon the use of local oxidation of silicon. Upon oxidation of a silicon substrate that is partially covered with a silicon nitride diffusion mask, lateral diffusion of oxidizing species underneath this mask still occurs. For wet and dry thermal oxidation of silicon, the silicon dioxide growing in the lateral direction is pushing the Si_3_N_4_ upward, which is visible as a bird’s beak in a cross-sectional view and as a ribbon in case of thick wet oxide layers upon use of sufficiently high processing temperatures.^40^ Such deformation and growth can negatively influence the formation of nanoscale wedges.^27,29^ In this work, the bird’s beak phenomenon might only play a role upon realization of the second-generation V-shaped nanogrooves (see Figure S1c for a schematic indication of the locations where bird’s beaks develop). In fact, upon realization of the second generation of nanogrooves by TMAH etching of silicon after selective removal of this Si_3_N_4_ mask, any effect of the bird’s beak will vanish: due to the non-zero etch rate of the Si {111} planes that confine the V-shaped nanogrooves, after TMAH etching the apices of the wedges are located below the silicon surface that was covered with Si_3_N_4_ (see Figure S1f). Therefore, the apices end up below the level at which the bird’s beak phenomenon could have occurred. Thus, this phenomenon has no (perceptible) negative geometrical effects on the realized self-aligned multiplied nanometric wedges.

To further enhance the resolution of the convex corner lithography as well as to increase the number of generations, it is important to work with a thinner mask layer. However, use of a thinner SiO_2_ layer (i.e., <8 nm) to reduce radius of curvature of concave corners is not an option, since this will lead to a too low thickness ratio between Si {111} and Si {100} (i.e., <1.5^41^), which is expected to hamper the convex corner lithography. The use of other material systems should therefore be explored, for example, replacing the oxidation by nitridation.

**Figure 6:**
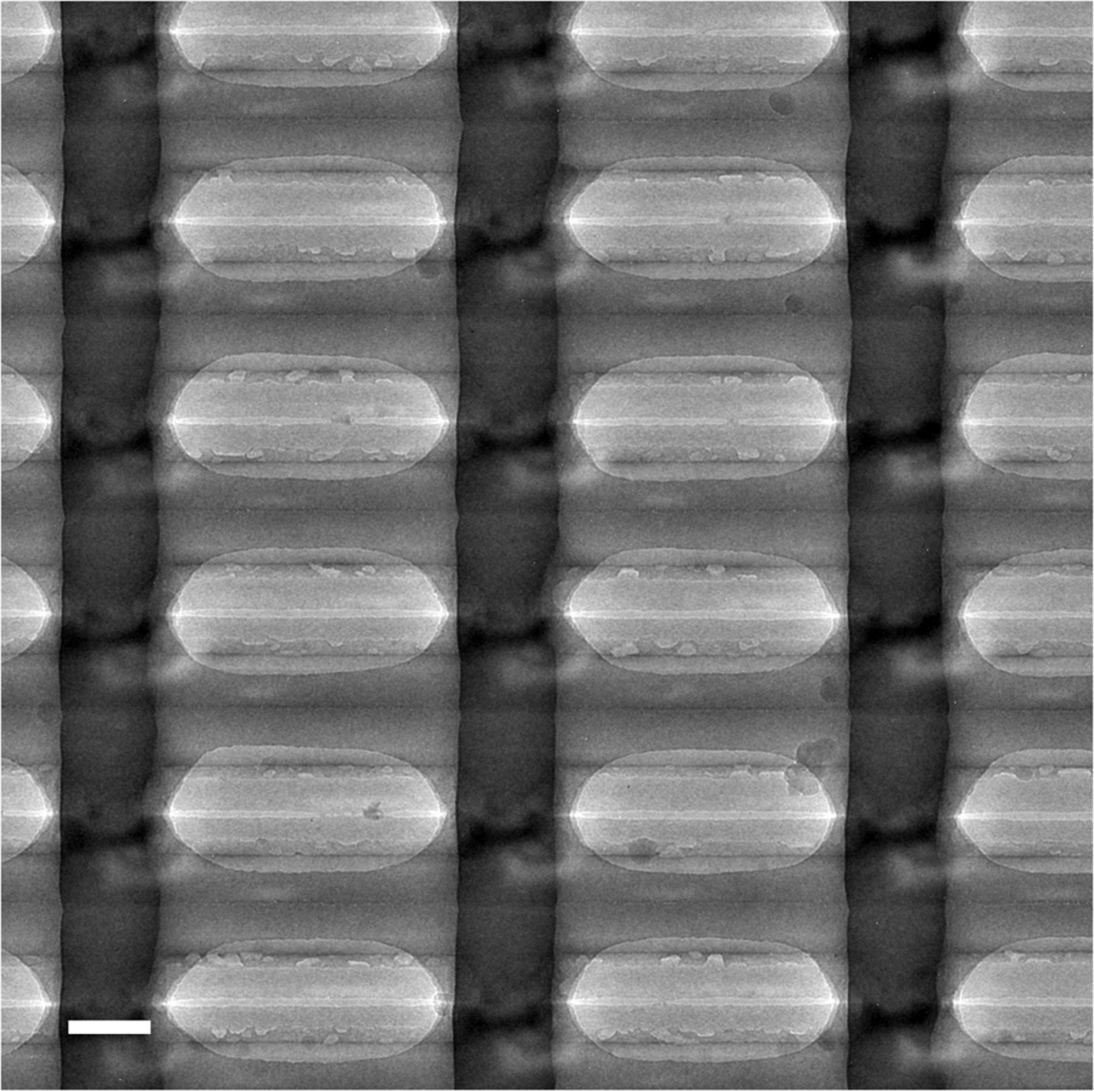
TEM image of part of the nanovalve array fabricated following the procedure described in ref (16). Scale bar represents 100 nm. The array is electron transparent because of its freestanding nature. The image confirms the (indirect) evidence from ref (16) that we have created arrays of flow-through nanoslits. Gap sizes can be estimated to be around 10 nm. Note that these flaps/slits where based on an initial silicon oxide thickness of 25 nm of which ∼10 nm remains after the convex corner lithography. In the current work, we have reduced this to 8 and 2 nm, respectively (see Figure 1).

The presented results of applying crystallographic nanolithography to convex corners of silicon wedges inspired to explore the possibility to create NEMS and other nanodevices at the apices of the high-density wedge array.^15,16^Figure S8 shows that arrays of freestanding silicon dioxide nanoflaps can be created, of which the “extruded” dimension of the freestanding flaps is confined by a displacement Talbot lithography (DTL) step (perpendicularly oriented with respect to the wedges) in combination with a hard mask to selectively apply the convex corner lithography only to parts of the wedges.^15^ In a follow-up study,^16^ such arrays of freestanding silicon dioxide flaps were combined with a strategy for controlled backside etching to create a flow-through device consisting of thousands of nanometric valves acting in parallel embedded in a mechanically stable membrane configuration. The massive parallel configuration enables “macroscopic” flow rates,^16^ while it is expected to maintain some of the nanoscopic features such as molecular flow regime and ultrafast mechanical response of the valves. Figure 6 shows a TEM image of a part of the array (top exposure) with an “as-fabricated” valve opening of around 10 nm, in correspondence with the (indirect) evidence from ref (16) that we have created these arrays of flow-through nanoslits. Note that these flaps/slits where based on an initial silicon oxide thickness of 25 nm of which ∼10 nm remains after the convex corner lithography. In the latest fabrication run detailed in the current manuscript, we have scaled down this to 8 and 2 nm, respectively.

## Conclusions and Outlook

Convex corner lithography is an emerging self-aligned nanometric patterning technique^15,16^ which now has been demonstrated to be employable down to the sub-10 nm scale. By repeatedly applying convex corner lithography in combination with wet anisotropic etching, double and quadruple feature densities were obtained. A wedge pitch of initially about 120 nm, as defined by the starting DTL step, could be reduced to about 30 nm on average by this procedure. After removal of the silicon dioxide mask, wedge arrays remained with sub-nm sharp apices. These sharp convex corners allowed for additional convex corner lithography to be applied, hence the opportunity for wedge self-aligned multiplication. Freestanding silicon dioxide mask features as thin as 1–2 nm could be conserved after etching a parallelepiped cavity.

Combining the approach shown in this work with the strategy introduced in ref (15) is expected to enable the (batch) fabrication of dense 2D arrays of nanodevices at the apices of the created wedges. First examples of such approach are shown in refs (16) and (42) where the density of devices can be further increased through the multiplication procedure. Therefore, nanometric Si {111} plane-terminated wedges combined with crystallographic nanolithography are promising templates for future multidomain nanosystems. These could include and combine NEMS and (opto)electronic devices, such as RF resonators, high-mobility channel transistors, III–V electronics, silicon photonics, and their monolithic integration.

## Supporting Information Available

Figure S1 (PDF)
Figure S2 (PDF)
Figure S3 (PDF)
Figure S4 (PDF)
Figure S5 (PDF)
Figure S6 (PDF)
Figure S7 (PDF)
Figure S8 (PDF)
Detailed clean room methods, process flow for initial wedge fabrication, SEM and TEM analysis of fabricated structure, 3D process flow description, uniformity check, and application in NEMS check valve (PDF)

## References

[ref1] Hunter W. R.; Holloway T. C.; Chatterjee P. K.; Tasch A. F. A new edge-defined approach for submicrometer MOSFET fabrication. IEEE Electron Device Lett. 1981, 2, 4–6. 10.1109/EDL.1981.25319.

[ref2] Gates B. D.; Xu Q.; Stewart M.; Ryan D.; Willson C. G.; Whitesides G. M. New approaches to nanofabrication: molding, printing, and other techniques. Chem. Rev. 2005, 105, 1171–1196. 10.1021/cr030076o. 15826012

[ref3] Auth C.; Aliyarukunju A.; Asoro M.; Bergstrom D.; Bhagwat V.; Birdsall J.; Bisnik N.; Buehler M.; Chikarmane V.; Ding G.; Fu Q.; Gomez H.; Han W.; Hanken D.; Haran M.; Hattendorf M.; Heusneer R.; Hiramatsu H.; Ho B.; Jaloviar S.; Jin I.; Joshi S.; Kirby S.; Kosaraju S.; Kothari H.; Leatherman G.; Lee K.; Leib J.; Madhavan A.; Marla K.; Meyer H.; Mule T.; Parker C.; Parthasarathy S.; Pelto C.; Pipes L.; Post I.; Prince M.; Rahman A.; Rajamani S.; Saha A.; Dacuna Santos J.; Sharma M.; Shin J.; Sinha P.; Smith P.; Sprinkle M.; St Amour A.; Staus C.; Suri R.; Towner D.; Tripathi A.; Tura A.; Ward C.; Yeoh A. In A 10 nm High Performance and Low-Power CMOS Technology Featuring 3rd Generation FinFET Transistors, Self-Aligned Quad Patterning, Contact Over Active Gate and Cobalt Local Interconnects, Proceedings of the 63rd IEEE International Electronic Devices Meeting (IEDM), San Franciso, CA, USA, December 2–6, 2017; pp 673–676.

[ref4] Xu P.; Chen Y.; Chen Y.; Miao L.; Sun S.; Kim S.-W.; Berger A.; Mao D.; Bencher C.; Hung R.; Ngai C. Sidewall spacer quadruple patterning for 15 nm half-pitch. Proc. SPIE , Optical Microlithography XXIV 2011, 7971, 79731Q 10.1117/12.881547.

[ref5] Liu W.; Wang J.; Xu X.; Zhao C.; Xu X.; Weiss P. S. Single-step dual-Layer photolithography for tunable and scalable nanopatterning. ACS Nano 2021, 15, 12180–12188. 10.1021/acsnano.1c03703. 34170108

[ref6] Lee K.; Kim D.; Yoon C.; Park T.; Han S.; Hwang Y.; Lee K.; Kang H.; Kim H. Self-aligned double patterning for active trim contacts with anisotropic pattern pitches in sub-20 nm dynamic random access memories. J. Micro/Nanolithogr., MEMS, MOEMS 2019, 18, 040501 10.1117/1.JMM.18.4.040501.

[ref7] Bae N.; Thibaut S.; Wada T.; Metz A.; Ko A.; Biolsi P. Advanced multiple patterning technologies for high density hexagonal hole arrays. Proc. SPIE, Advanced Etch Technology and Process Integration for Nanopatterning X 2021, 11615, 116150B 10.1117/12.2584594.

[ref8] Sarajlic E.; Berenschot E.; Krijnen G.; Elwenspoek M. In Fabrication of 3D Nanowire Frames by Conventional Micromachining Technology, Proceedings of the 13th International Conference on Solid-State Sensors, Actuators and Microsystems (Transducers), Seoul, South Korea, June 5–9, 2005; pp 27–30.

[ref9] Berenschot E.; Tas N. R.; Jansen H. V.; Elwenspoek M. In 3D-Nanomachining Using Corner Lithography, Proceedings of the 3rd IEEE International Conference on Nano/Micro Engineered and Molecular Systems, Sanya, China, January 6–9, 2008; pp 729–732.

[ref10] Berenschot E. J. W.; Burouni N.; Schurink B.; Van Honschoten J. W.; Sanders R. G. P.; Truckenmuller R.; Jansen H. V.; Elwenspoek M. C.; Van Apeldoorn A. A.; Tas N. R. 3D nanofabrication of fluidic components by corner lithography. Small 2012, 8, 3823–3831. 10.1002/smll.201201446. 22907803

[ref11] Berenschot E. J. W.; Jansen H. V.; Tas N. R. Fabrication of 3D fractal structures using nanoscale anisotropic etching of single crystalline silicon. J. Micromech. Microeng. 2013, 23, 055024 10.1088/0960-1317/23/5/055024.

[ref12] Zhao Y.; Berenschot E.; Jansen H.; Tas N.; Huskens J.; Elwenspoek M. C. Multi-silicon ridge nanofabrication by repeated edge lithography. Nanotechnology 2009, 20, 315305 10.1088/0957-4484/20/31/315305. 19597243

[ref13] Zhao Y.; Berenschot E.; Jansen H.; Tas N.; Huskens J.; Elwenspoek M. Sub-10 nm silicon ridge nanofabrication by advanced edge lithography for NIL applications. Microelectron. Eng. 2009, 86, 832–835. 10.1016/j.mee.2008.11.067.

[ref14] Berenschot J. W.; Tas N. R.; Jansen H. V.; Elwenspoek M. Chemically anisotropic single-crystalline silicon nanotetrahedra. Nanotechnology 2009, 20, 475302 10.1088/0957-4484/20/47/475302. 19858560

[ref15] Berenschot E.; Deenen C.; Tiggelaar R.; Gardeniers H.; Tas N. In Waferscale Fabrication of Nanogaps by Convex Corner Lithography, Proceedings of the 6th International Workshop on Nanotechnology and Application, Phan Thiet, Vietnam, November 8–11, 2017.

[ref16] Van Kampen C. P.; Berenschot E. J.; Burger G.-J.; Tiggelaar R. M.; Sanders R. G. P.; Gardeniers H. J. G. E.; Tas N. R. In Massive Parallel NEMS Flow Restriction Fabricated Using Self-Aligned 3D-Crystallographic Nanolithography, Proceedings of the 33rd International Conference on Micro Electro Mechanical Systems (MEMS), Vancouver, Canada, January 18–22, 2020; pp 1106–1109.

[ref17] Kao D.-B.; McVittie J. P.; Nix W. D.; Saraswat K. C. Two-dimensional thermal oxidation of silicon – I. Experiments. IEEE Trans. Electron Devices 1987, 34, 1008–1017. 10.1109/T-ED.1987.23037.

[ref18] Marcus R. B.; Sheng T. T. The oxidation of shaped silicon surfaces. J. Electrochem. Soc. 1982, 129, 1282–1289. 10.1149/1.2124118.

[ref19] Gray H. F.; Campisi G. J.; Greene R. F. In A Vacuum Field Effect Transistor Using Silicon Field Emitter Arrays., Technical Digest of the 32nd International Electron Devices Meeting (IEDM), Los Angeles, CA, USA, December 7–10, 1986; pp 776–778.

[ref20] Campisi G. J.; Gray H. F. Microfabrication of field emission devices for vacuum integrated circuits using orientation dependent etching. Mat. Res. Soc. Symp. Proc. 1986, 76, 67–72. 10.1557/PROC-76-67.

[ref21] Gray H. F.; Shaw J. L. In Point and Wedge Tungsten-on-Silicon Field Emitter Arrays, Technical Digest of the 37th International Electron Devices Meeting (IEDM), Washington, CA, USA, December 8–11, 1991; pp 221–224.

[ref22] Jones G. W.; Sune C. T.; Gray H. F. Silicon field emission transistors and diodes, IEEE Trans. Components. Hybrids Manuf. Technol. 1992, 15, 1051–1055. 10.1109/33.206930.

[ref23] Hashiguchi G.; Sakamoto H.; Kanazawa S.; Mimura H. Wedge-shaped silicon emitter fabricated by new method. Jpn. J. Appl. Phys. 1993, 32, 6291–6292. 10.1143/JJAP.32.6291.

[ref24] Hashiguchi G.; Sakamoto H.; Kanazawa S.; Mimura H. Fabrication and emission characteristics of new wedge-shaped silicon emitters. Appl. Surf. Sci. 1994, 76/77, 41–46. 10.1016/0169-4332(94)90321-2.

[ref25] Hashiguchi G.; Mimura H. Fabrication of silicon quantum wires using separation by implanted oxygen wafer. Jpn. J. Appl. Phys. 1994, 33, L1649–L1650. 10.1143/JJAP.33.L1649.

[ref26] Hashiguchi G.; Mimura H. New fabrication methods and electrical characteristics of conical silicon field emitters. Jpn. J. Appl. Phys. 1995, 34, 1493–1497. 10.1143/JJAP.34.1493.

[ref27] Chen S.; Bomer J. G.; Van der Wiel W. G.; Carlen E. T.; Van den Berg A. Top-down fabrication of sub-30 nm monocrystalline silicon nanowires using conventional microfabrication. ACS Nano 2009, 3, 3485–3492. 10.1021/nn901220g. 19856905

[ref28] Ribbing C.; Cederström B.; Lundqvist M. Microfabrication of saw-tooth refractive x-ray lenses in low-Z materials. J. Micromech. Microeng. 2003, 13, 714–720. 10.1088/0960-1317/13/5/325.

[ref29] Jin M.; Zhu Y.; Van den Berg A.; Zhang Z.; Zhou G.; Shui L. Wafer-scale fabrication of high-density nanoslit arrays for surface-enhanced Raman spectroscopy. Nanotechnology 2016, 27, 49LT01 10.1088/0957-4484/27/49/49LT01. 27831932

[ref30] Wilbers J. G. E.; Berenschot J. W.; Tiggelaar R. M.; Dogan T.; Sugimura K.; Van der Wiel W. G.; Gardeniers J. G. E.; Tas N. R. 3D-fabrication of tunable and high-density arrays of crystalline silicon nanostructures. J. Micromech. Microeng. 2018, 28, 044003 10.1088/1361-6439/aaab2d.

[ref31] Gong Y.; Dai P.; Gao A.; Li T.; Zhou P.; Wang Y. *In situ* nanoscale refinement by highly con-trollable etching of the (111) silicon crystal plane and its influence on the enhanced electrical property of a silicon nanowire. J. Semicond. 2011, 32, 123003 10.1088/1674-4926/32/12/123003.

[ref32] Kao D.-B.; McVittie J. P.; Nix W. D.; Saraswat K. C. Two-dimensional thermal oxidation of silicon – II. Modeling stress effects in wet oxides. IEEE Trans. Electron Devices 1988, 35, 25–37. 10.1109/16.2412.

[ref33] Krzeminksi C. D.; Han X.-L.; Larrieu G. Understanding of the retarded oxidation effects in silicon nanostructures. Appl. Phys. Lett. 2012, 100, 263111 10.1063/1.4729410.

[ref34] Irene E. A. Silicon oxidation studies: A revised model for thermal oxidation. J. Appl. Phys. 1983, 54, 5416 10.1063/1.332722.

[ref35] Irene E. A.; Tierney E.; Angilello J. A viscous flow model to explain the appearance of high density thermal SiO_2_ at low oxidation temperatures. J. Electrochem. Soc. 1982, 129, 2594–2597. 10.1149/1.2123617.

[ref36] Falk M. L.; Langer J. S. Deformation and failure of amorphous, solidlike materials. Annu. Rev. Condens. Matter Phys. 2011, 2, 353–373. 10.1146/annurev-conmatphys-062910-140452.

[ref37] Massoud H. Z.; Plummer J. D.; Irene E. A. Thermal oxidation of silicon in dry oxygen: accurate determination of the kinetic rate constants. J. Electrochem. Soc. 1985, 132, 1745–1753. 10.1149/1.2114204.

[ref38] Massoud H. Z.; Plummer J. D. Analytical relationship for the oxidation of silicon in dry oxygen in the thin-film regime. J. Appl. Phys. 1987, 62, 3416–3423. 10.1063/1.339305.

[ref39] Appels J. A.; Kooi E.; Paffen M. M.; Schatorjé J. J. H.; Verkuylen W. H. C. G. Local oxidation of silicon and its application in semiconductor-device technology. Philips Res. Rep. 1970, 25, 118–132.

[ref40] Kooi E.; Van Lierop J. G.; Appels J. A. Formation of silicon nitride at a Si-SiO_2_ interface during local oxidation of silicon and during heat-treatment of oxidized silicon in NH_3_ gas. J. Electrochem. Soc. 1976, 123, 1117–1120. 10.1149/1.2133008.

[ref41] Momose H. S.; Ohguro T.; Kojima K.; Nakamura S.; Toyoshima Y. 1.5-nm gate oxide CMOS on (110) surface-oriented Si substrate. IEEE Trans. Electron. Devices 2003, 50, 1001–1008. 10.1109/TED.2003.812085.

[ref42] Kim M.-H.; Cho S.; Park B-G. Nanoscale wedge resistive-switching synaptic device and experimental verification of vector-matrix multiplication for hardware neuromorphic application. Jpn. J. Appl. Phys. 2021, 60, 050905 10.35848/1347-4065/abf4a0.

