# Supplementary figures and images for: Self-Aligned Crystallographic Multiplication of Nanoscale Silicon Wedges for High-Density Fabrication of 3D Nanodevices
Source: ACS Appl Nano Mater. 2022 Oct 12;5(10):15847–54. doi: 10.1021/acsanm.2c04079 (PMC9623545; doi:10.1021/acsanm.2c04079)

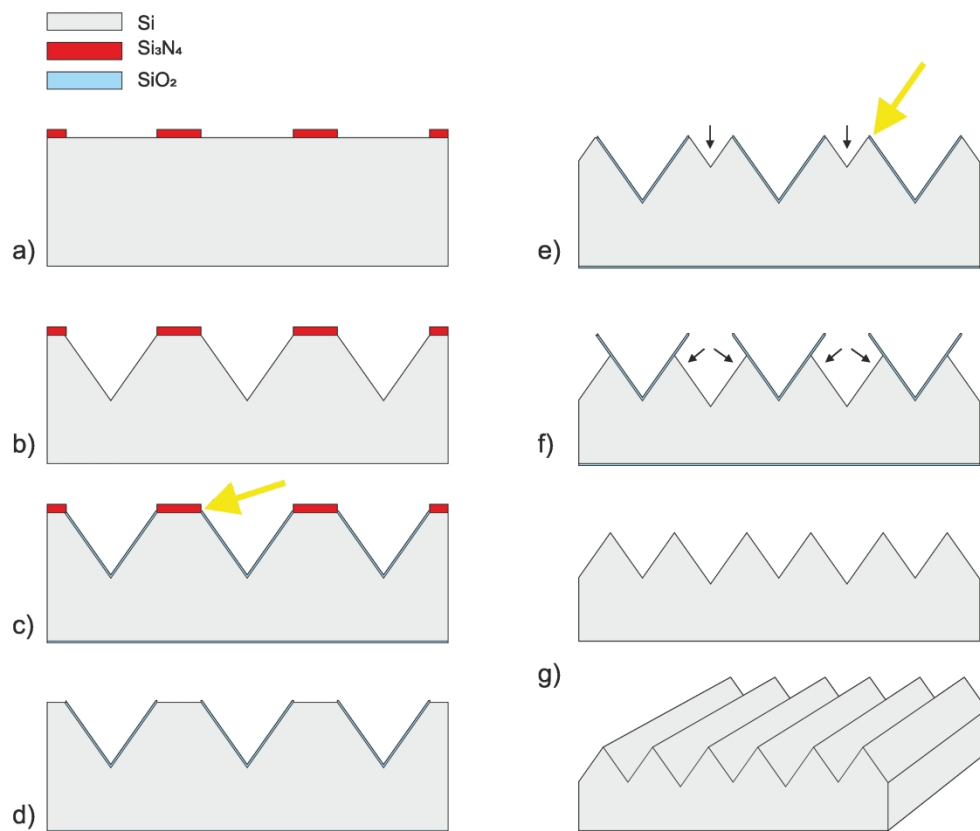

271x236mm (299 x 299 DPI)

Supplement: Supplementary file 1 — an2c04079_si_001.pdf [file an2c04079_si_001.pdf]

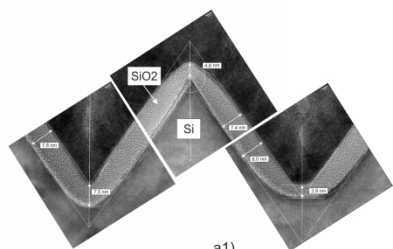

a1)

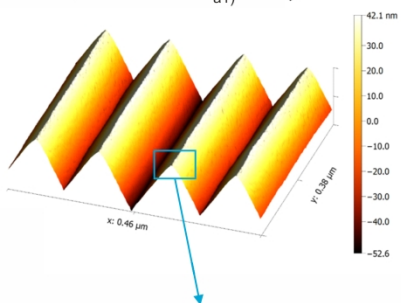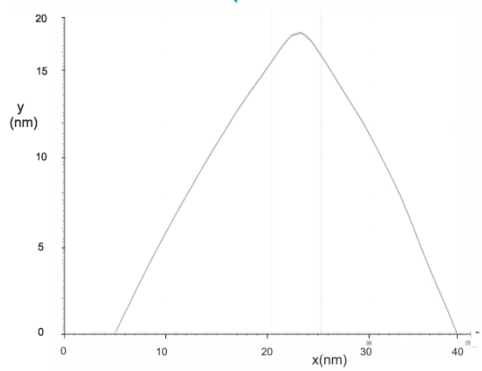

a2)

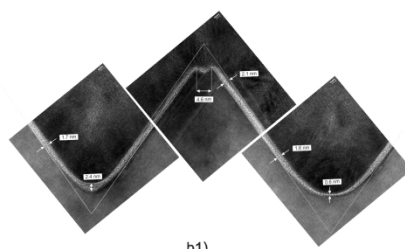

b1)

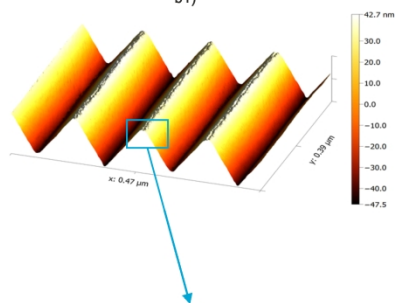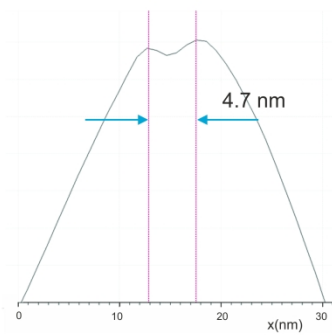

b2)

Supplement: Supplementary file 2 — an2c04079_si_002.pdf [file an2c04079_si_002.pdf]

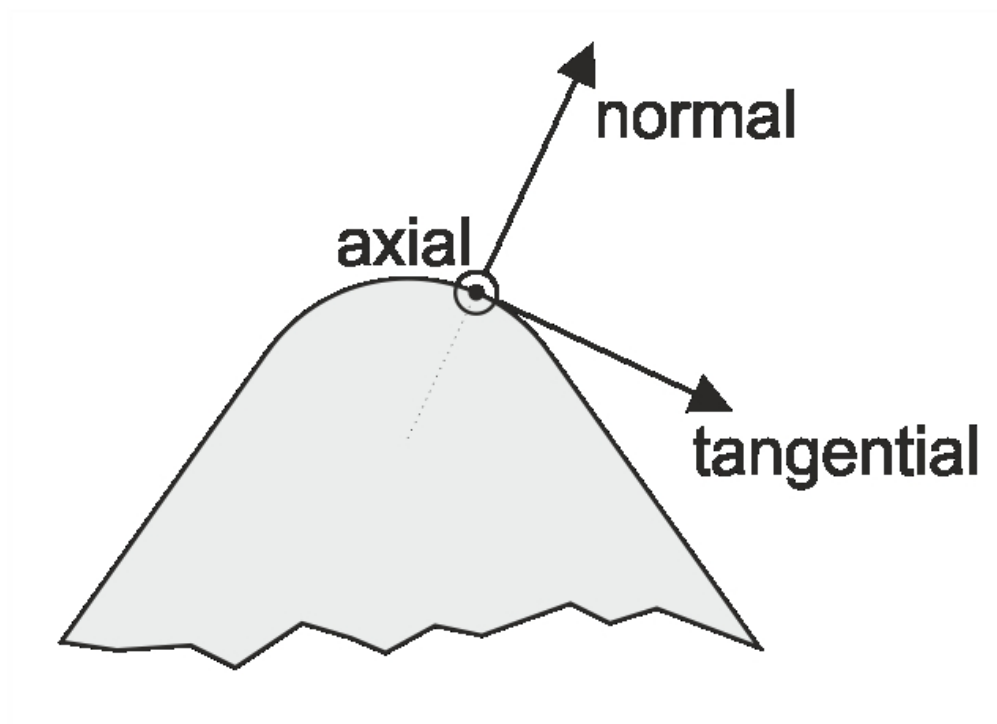

Supplement: Supplementary file 3 — an2c04079_si_003.pdf [file an2c04079_si_003.pdf]

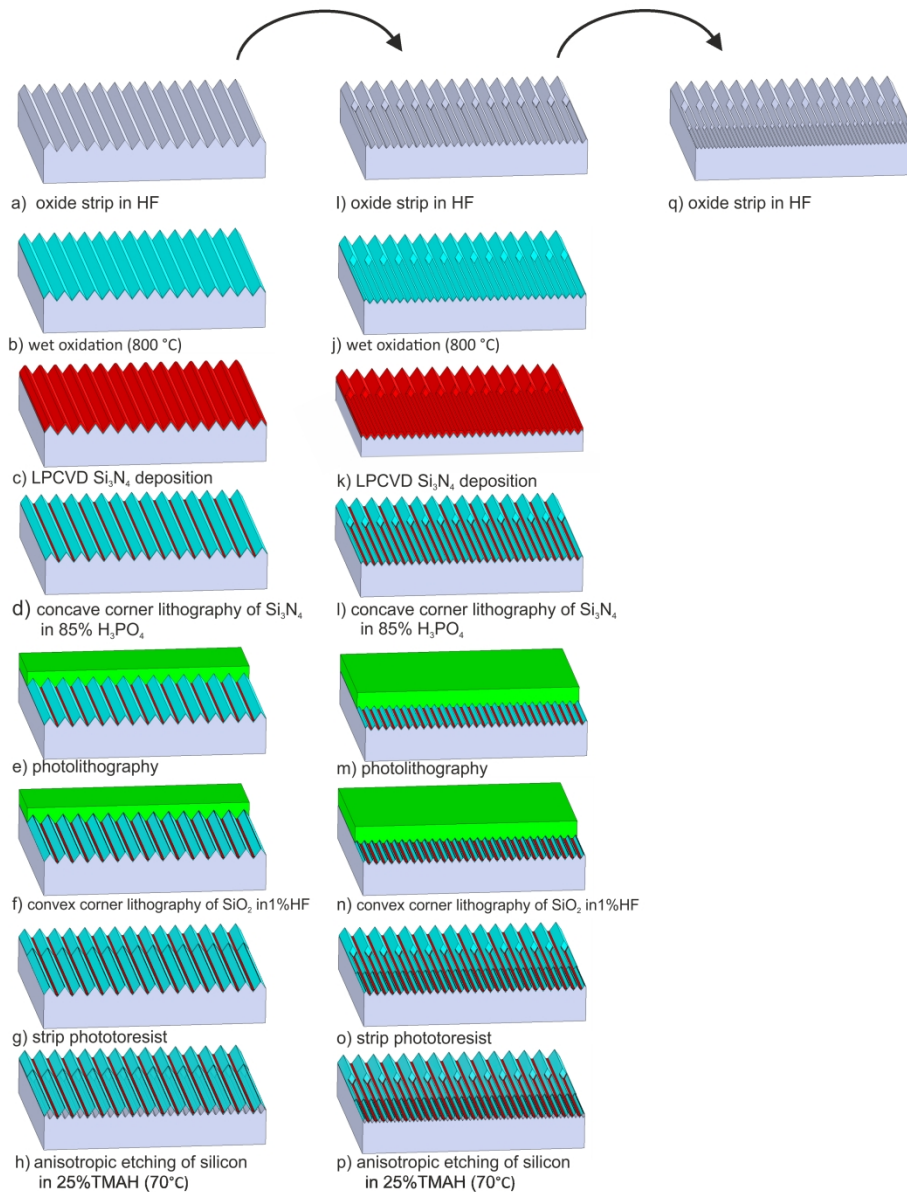

Supplement: Supplementary file 4 — an2c04079_si_004.pdf [file an2c04079_si_004.pdf]

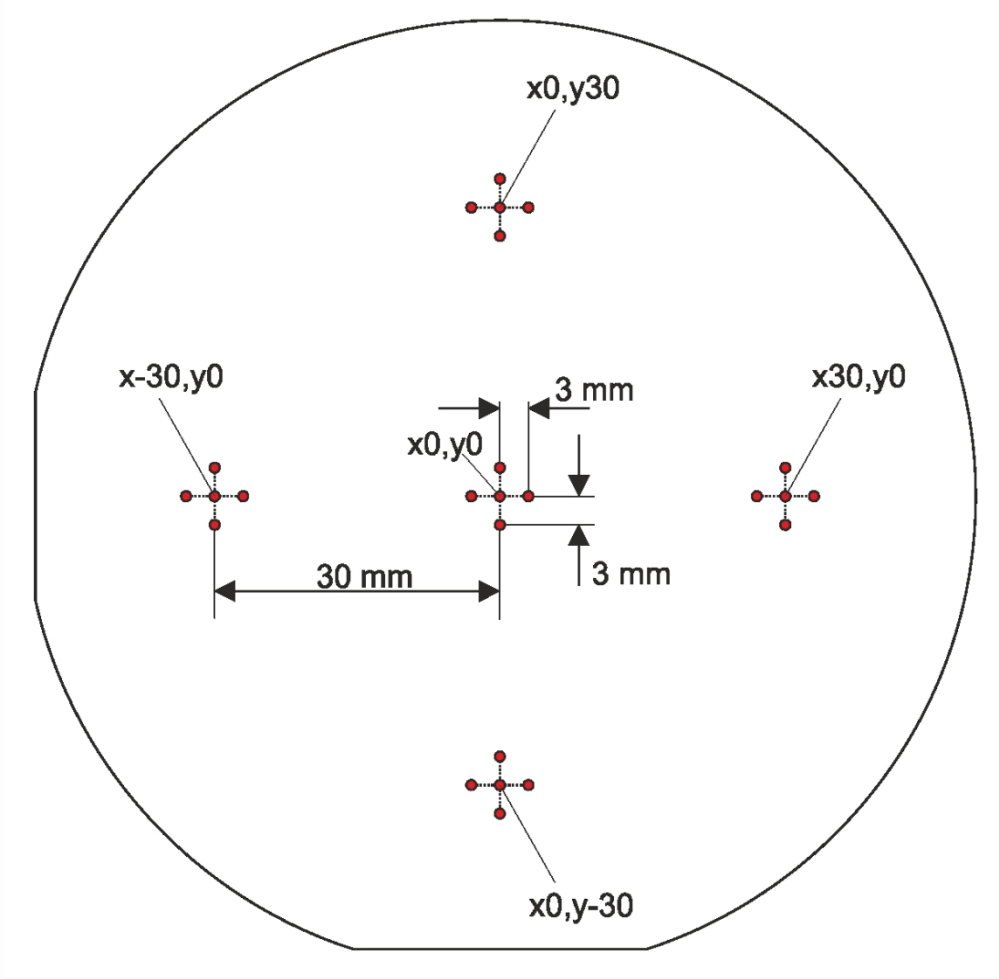

Supplement: Supplementary file 5 — an2c04079_si_005.pdf [file an2c04079_si_005.pdf]

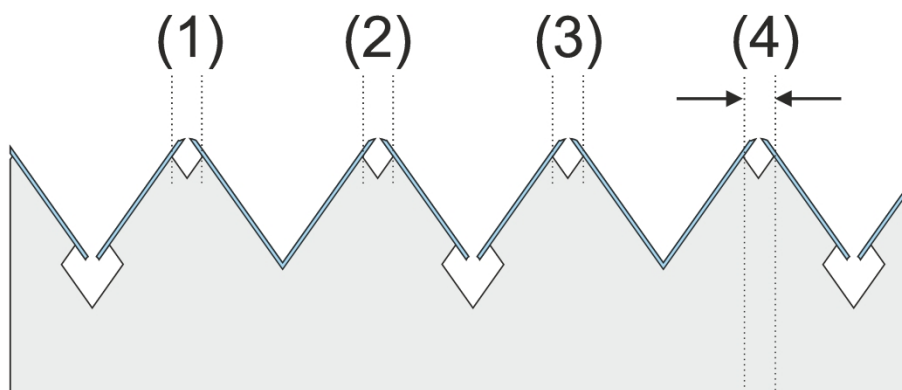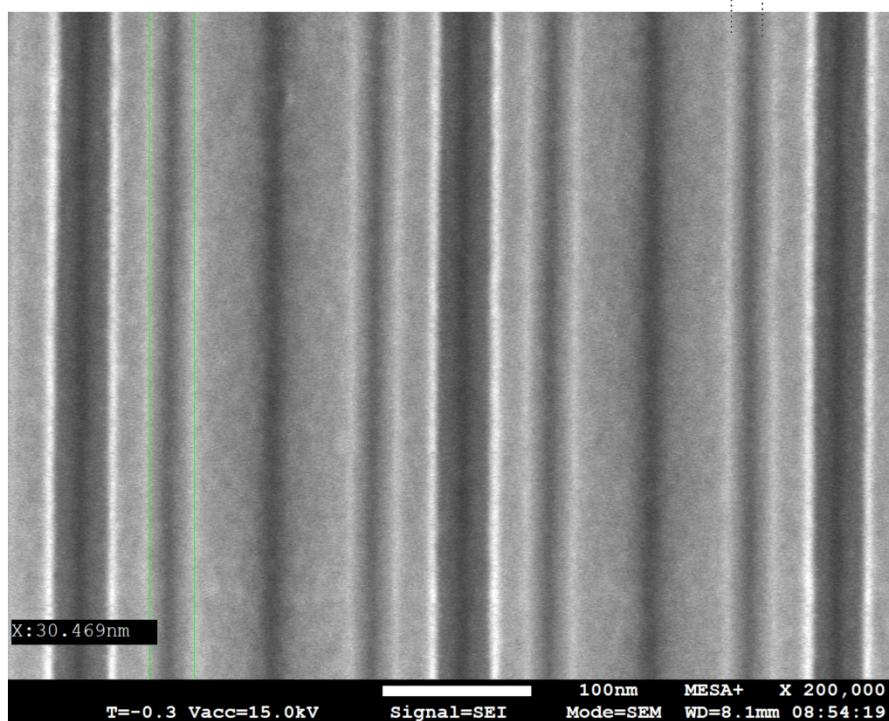

Supplement: Supplementary file 6 — an2c04079_si_006.pdf [file an2c04079_si_006.pdf]

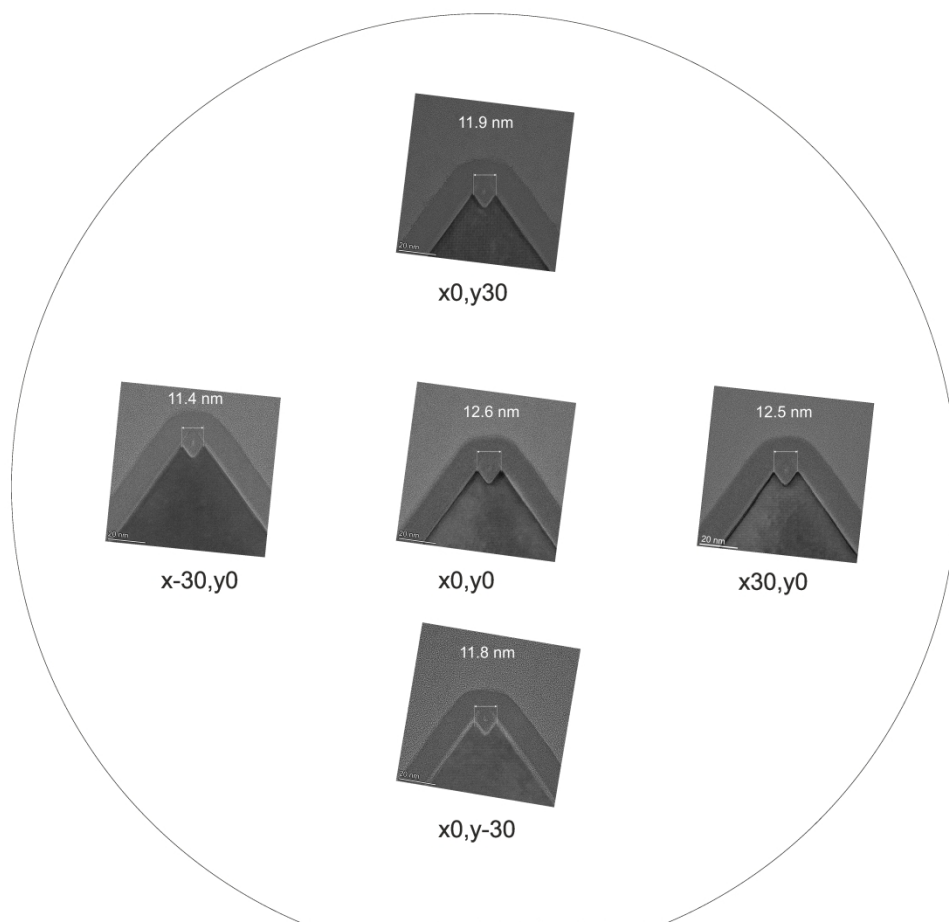

Supplement: Supplementary file 7 — an2c04079_si_007.pdf [file an2c04079_si_007.pdf]

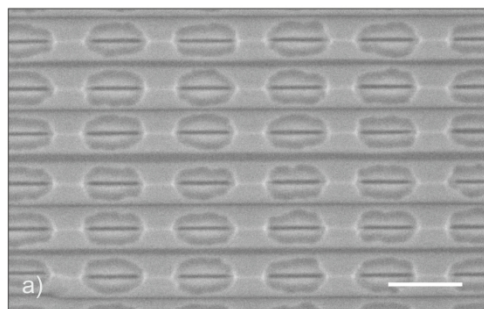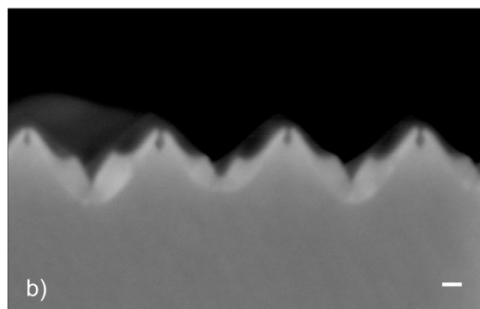

Supplement: Supplementary file 8 — an2c04079_si_008.pdf [file an2c04079_si_008.pdf]
