# Supplementary material for: Self-Aligned Crystallographic Multiplication of Nanoscale Silicon Wedges for High-Density Fabrication of 3D Nanodevices
Source: ACS Appl Nano Mater. 2022 Oct 12;5(10):15847–54. doi: 10.1021/acsanm.2c04079 (PMC9623545; doi:10.1021/acsanm.2c04079)
Supplement: Supplementary file 9 — an2c04079_si_009.pdf [file an2c04079_si_009.pdf]

SUPPORTING INFORMATION

Self-Aligned Crystallographic Multiplication of Nanoscale Silicon Wedges for High-Density Fabrication of 3D Nanodevices

Erwin Berenschot,*§ Roald M. Tiggelaar,† Bjorn Borgelink,§ Chris van Kampen,§ Cristian S. Deenen,§ Yasser Pordeli,§ Haye Witteveen, § Han J. G. E. Gardeniers § and Niels R. Tas*§

§Mesoscale Chemical Systems, MESA+ Institute, University of Twente, Drienerlolaan 5, 7522 NB Enschede, The Netherlands †NanoLab Cleanroom, MESA+ Institute, University of Twente, Drienerlolaan 5, 7522 NB Enschede, The Netherlands

Corresponding Authors
*E-mail: j.w.berenschot@utwente.nl
*E-mail: n.r.tas@utwente.nl

1. Detailed clean room fabrication methods for the process illustrated in figure S1 and figure 3

Fabrication of nano-wedges is done in highly boron doped (100)-oriented silicon wafers (100 mm diameter, 525 µm thick, resistivity 0.01-0.025 Ωcm, one-side polished; Okmetic, Finland). A schematic representation of the experimental procedure is given in fig. S1 (nano-wedge fabrication and pattern doubling) and fig. 3 (self-aligned multiplication of nano-wedges by means of crystallographic nanolithography). The detailed experimental procedure is as follows:

Prior to low-pressure chemical vapor deposition (LPCVD; Tempress horizontal diffusion system, type TS6604, 800°C, 200 mTorr, 22 sccm SiH_2Cl_2, 66 sccm NH_3) of stoichiometric silicon nitride (Si_3N_4), the substrates were cleaned with fuming 99% nitric acid (HNO_3) (2x 5 min. – KMG Ultra Pure Chemicals) and boiling 69% HNO_3 (10 min. – BASF) after which the silicon oxide layer was removed with 1% hydrofluoric acid (HF) (1 min. immersion (at room temperature) – Technic). The deposited silicon nitride layer thickness was 13.0 ± 0.1 nm (deposition time 17 min.), as determined with ellipsometry (Woollam M-2000UI). Standard UV-lithography (resist Olin 907-17 – FujiFilm) in combination with etching in buffered hydrofluoric acid (BHF) (28 min. (at room temperature) – Technic) was used to define so-called Vangbo structures in the Si_3N_4 film. This pattern was transferred into the silicon using 25 wt.% tetramethylammonium hydroxide (TMAH) (temperature 70 °C; etch time 34 min. – Merck). Vangbo structures were utilized to find the exact crystallographic orientation of silicon wafers [S1] and these structures were applied for precise alignment (i.e. 0.05° accuracy) of mask patterns to the <110>-direction of the silicon crystal. More specific, a phase-shift line mask (linewidth: 250 nm, pitch: 500 nm) was aligned to the <110>-direction of a (100)-Si wafer – to minimize/avoid the occurrence of lattice kinks on {111}-Si planes (and subsequently at the apices) – by employing the advanced interference lithography technique DTL (PhableR 100C, Eulitha, Switzerland) [S2 – S4]. After this DTL-step, a line pattern with a resist linewidth of 117 nm and a spacer width of 130 nm was present in a layer of PFI88 photoresist (1:1 PFI:PGMEA (propylene glycol monomethyl ether acetate – Sumitomo Chemical Co., Ltd.)) [S5]. This pattern was transferred - at a 1:1 ratio - into the underlying bottom anti-reflection coating (BARC; AZ Barli-II 200) with reactive ion etching (RIE) in a directional N_2 plasma (50 mTorr, 50 sccm N_2, 8.5 min; home-built system) [S3, S5]. Subsequently, with RIE the Si_3N_4 was selectively and directionally etched (10 mTorr, 25 sccm CHF_3, 5 sccm O_2, 25W, 40 sec; home-built system) using the PR/BARC coating as mask. Afterwards the mask was removed with an O_2-plasma (Tepla 300E, 30 min.) followed by immersion in 100% HNO_3 (2x 5 min.) and removal of the interfacial SiON/oxide interface with BHF (2 min. (at room temperature)). The patterned Si_3N_4 served as hard mask for anisotropic etching in room-temperature 20 wt.% potassium hydroxide (KOH) (etch time 5.5 min. – Merck). This yielded the first generation of V-shaped nano-grooves, with a width of ca. 130 nm and in-between the V-grooves flat spacers of ca. 117 nm width. KOH-etching was followed by RCA-2 cleaning for 15 min to remove alkali-residue. RCA-2 is a mixture (80 °C) of 36% hydrochloric acid (HCl – BASF), 31% hydrogen-peroxide (H_2O_2 – BASF) and demineralized water (DI water) in a volumetric ration of 1:1:5.

The next step was doubling of the periodicity of the V-grooves, i.e. removal of the flat spacers between the 1st generation V-grooves (see fig. S1 for the general process). This was accomplished with Local Oxidation of Silicon (LOCOS) in combination with a second anisotropic etching, a procedure of which details are described by Wilbers et.al [S5]. In brief, wet oxidation at 800 °C (15 min.; Tempress) yielded a SiO_2-layer of 7.9 ± 0.4 nm on Si-{111} (i.e. the walls of the V-grooves; for comparison: this oxidation step yields 5.7 ± 0.3 nm SiO_2 on Si-{100}), whereas the Si_3N_4-covered (100)-Si surface was only slightly

oxidized. A dip in 1% HF (15 sec (at room temperature)) was applied to remove this oxide from the Si_3N_4, followed by selective removal of the Si_3N_4 layer in 85% phosphoric acid (H_3PO_4) (140 °C, 18 min. – BASF). A combination of an UV-ozone treatment (5 min.) and immersion in 1% HF (11 sec. (at room temperature)) was used to remove the interfacial oxide underneath the Si_3N_4 and resulted in hydrophobic silicon. The exposed Si was etched in 25 wt.% TMAH (70 °C), in which the etch rates of Si-{100} and Si-{111} are ca. 300 nm/min and 9.7 nm/min, respectively. An etch time of 6 min. resulted in a width of ca. 125.0 ± 2.0 nm for both the 1st and 2nd generation V-grooves: due to the (slow) etching of Si-{111} the initial width (of ca. 130 nm) of 1st generation V-grooves is reduced to ca. 125 nm. A 50%-50% duty-cycle of the doubled V-groove pattern – defined as the ratio of the widths of the 1st and 2nd generation V-grooves after the KOH and TMAH etching steps – can thus be accomplished via optimization of the etching time in TMAH [30]. After the TMAH etch step the SiO_2 layer was removed with 50% HF (1 min. – BASF).

The periodicity of the realized V-groove pattern was doubled via a combination of SiO_2-based convex CL and Si_3N_4-based concave CL. First, the substrate was wet thermally oxidized for 15 min. at 800 °C (7.9 ± 0.4 nm on Si-{111}). On top of this layer a film of LPCVD Si_3N_4 was deposited (17 min, 13.1 ± 0.1 nm) onto which corner lithography was applied: the Si_3N_4 layer was isotropically etched for 18 min. in hot H_3PO_4 (85%, 140 °C), after which only Si_3N_4 was left in the concave corners of the V-grooves. Subsequently, convex corner lithography was applied to the SiO_2 layer: isotropic thinning in 1% HF for 67 seconds resulted in exposed Si at the convex corners, whereas the Si-{111}-walls were still covered with SiO_2 and the concave corners with SiO_2 and Si_3N_4. With TMAH (25 wt.%, 70 °C) the silicon at the apices was etched for 5.5 min, resulting in a 3rd generation V-grooves with a width of 61.3 ± 2.0 nm. After cleaning in HNO_3 (fuming 99% and boiling 69%) the remaining layers of SiO_2 and Si_3N_4 were simultaneously stripped in 50% HF (etch time 2 min. at room temperature).

Finally, a 3rd doubling of the periodicity of the groove-pattern was realized. The applied procedure was identical to the above-mentioned corner lithography sequence consisting of SiO_2-convex and Si_3N_4-concave corner processing, with the exception that the etch time in TMAH was 105 seconds. By means of this procedure a 4th generation V-shaped nano-grooves was created, with a width of 30.6 ± 5.0 nm.

When the above-mentioned V-groove periodicity doubling procedure based on crystallographic nanolithography is combined with standard UV-lithography (Olin 907-17 photoresist) in an orthogonal fashion, i.e. the UV-based mask pattern is 90° rotated with respect to the length-direction of the wedges, it is possible to realize adjacent areas of wedges of which the periodicity varies by a factor 2.

2. General process of V-groove pattern doubling

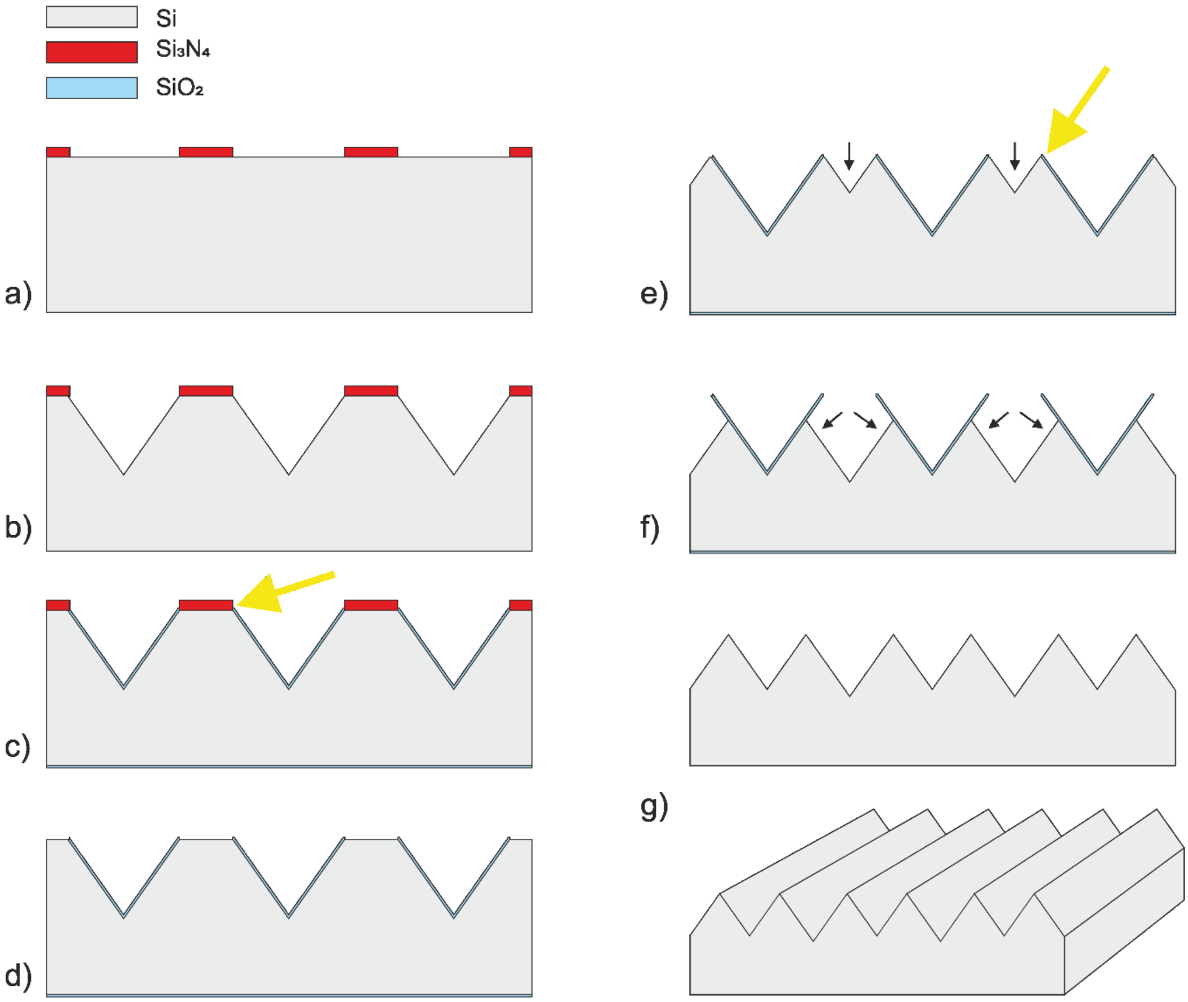

Figure S1. General process to create V-groove pattern doubling: a) silicon nitride line patterning, b) anisotropic silicon etching, c) local oxidation of silicon (LOCOS), d) selective removal of silicon nitride in phosphoric acid, e) fast anisotropic etching Si(100) followed by f) slow anisotropic etching Si(111) planes and tuning of the dutycycle, g).removal of the oxide with HF. The arrows indicate the location where bird's beaks develop during LOCOS. For full process details, see the Methods section in the main manuscript.

3. Analysis of the TEM and AFM data obtained of at least one period of the etched silicon nano-wedges

In figure S2 TEM and AFM images are shown of the convex as well as the concave corners of a full nano-wedge period during two key steps shown in fig. 1 of the main text.

Fig. S2a1) shows in high detail the grown oxide on the convex and concave corners. The silicon substrate was wet thermally oxidized for 15 min. at 800 °C (7.9 ± 0.4 nm on Si(111), 5.7 ± 0.3 nm on Si(100)). The reason that 2 concave corners are shown is that these corners have a different radius of curvature because of the difference in number of previous oxidation steps. The concave corner on the left is oxidized for the first time, whereas the one on the right is oxidized twice (using the same oxidation settings and an HF strip in between). A first oxidation step with the above-mentioned settings results in a conformal concave oxide thickness (ca. 7.5 nm), whereas the second oxidation is much thinner at the concave corner (ca. 3.9 nm). Fig. S2a2) shows the AFM result of the formed nano-wedges.

Figs. S2b1) and b2) correspond to the anisotropic etching step shown in fig. 1c) (main text). In the AFM image clearly the start of the anistropic etching can be identified, beginning exclusively at the convex corner (in 20 wt% KOH at room temperature).

The used AFM system is a Bruker Fast Scan / ICON operated in the ScanAsyst-air mode. The used tips are SAA-HPI-SS (Bruker). Tip radius (nom) = 1 nm, Tip radius (max) = 2 nm.

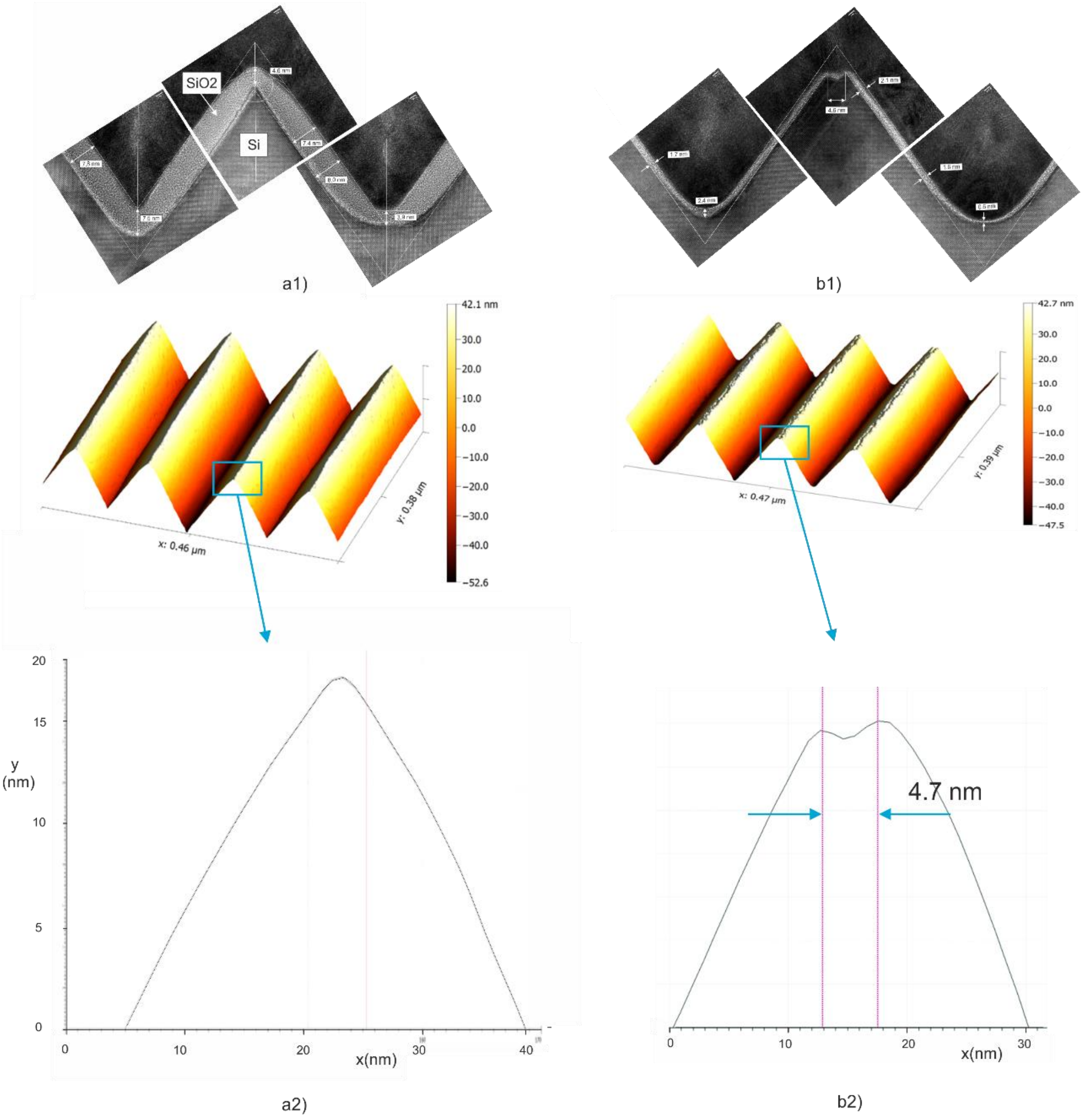

Figure S2. TEM and AFM images of key processing steps as shown in fig. 1 of the main text.

4. Definition of directions for the discussion on stresses developing near corners during thermal oxidation

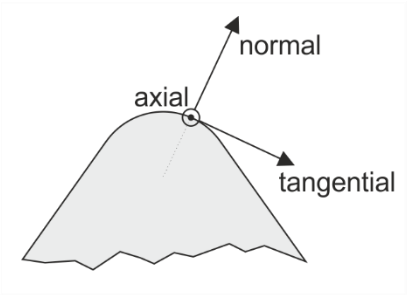

Figure S3. Definition of directions of the stress components developing near the apex of a wedge during thermal oxidation.

5. 3D representation of the double repeated self-aligned multiplying wedge nanomachining procedure

In fig. S4 a 3D representation is given of the (double repeated) self-aligned multiplying wedge nanomachining procedure. At each subfigure a short description of the process step is given. A detailed textual description of the fabrication sequence is provided in SI section 1.

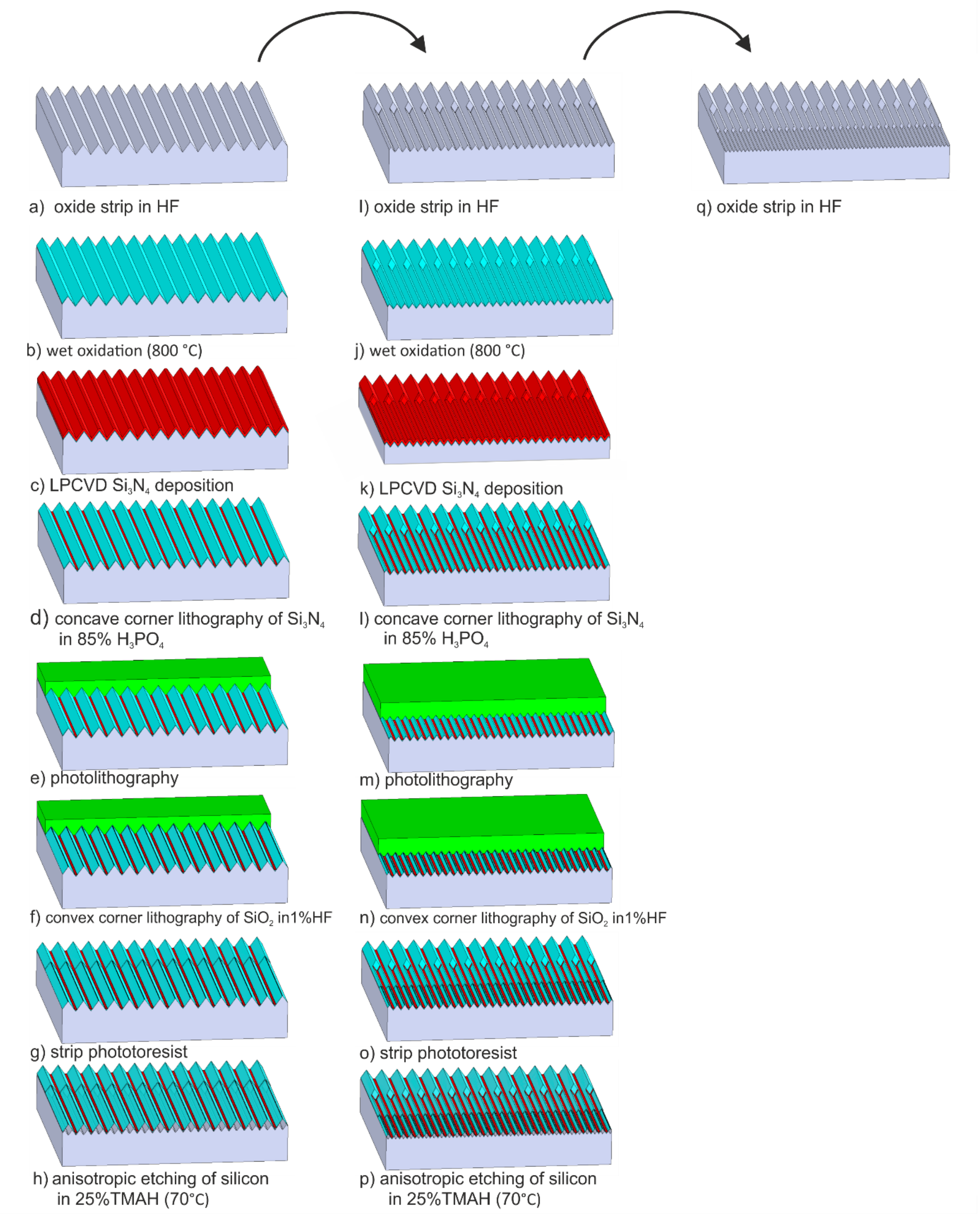

Figure S4. 3D representation of the (double repeated) self-aligned multiplying wedge nanomachining procedure.

6. Uniformity of double repeated self-aligned multiplying wedge nano-machining procedure with tuned 50% duty-cycle

The uniformity of key steps of the self-aligned multiplying wedge nanomachining procedure was first analyzed based on a separate run which includes all steps needed for a single multiplication step (convex corner lithography + anisotropic etching) and which omits the concave (protective corner lithography). On 5x5 locations, distributed across a substrate as indicated in figure S5, SEM images were recorded. Based on these images (a typical example is shown in figure S6), the pitch of the wedges was determined (after 240 sec of TMAH etching). Table S1 shows the obtained pitches at the various locations (coordinates shown in fig. S5); it is noted that the width values in the first column were determined with the software of the SEM, whereas the values in columns 2-4 were obtained by image analysis with CorelDRAW X7. The average (i.e., arithmetic mean) wedge pitch across a substrate is 31.0 nm width a σ-value (i.e., SD_N) of 1.1 nm.

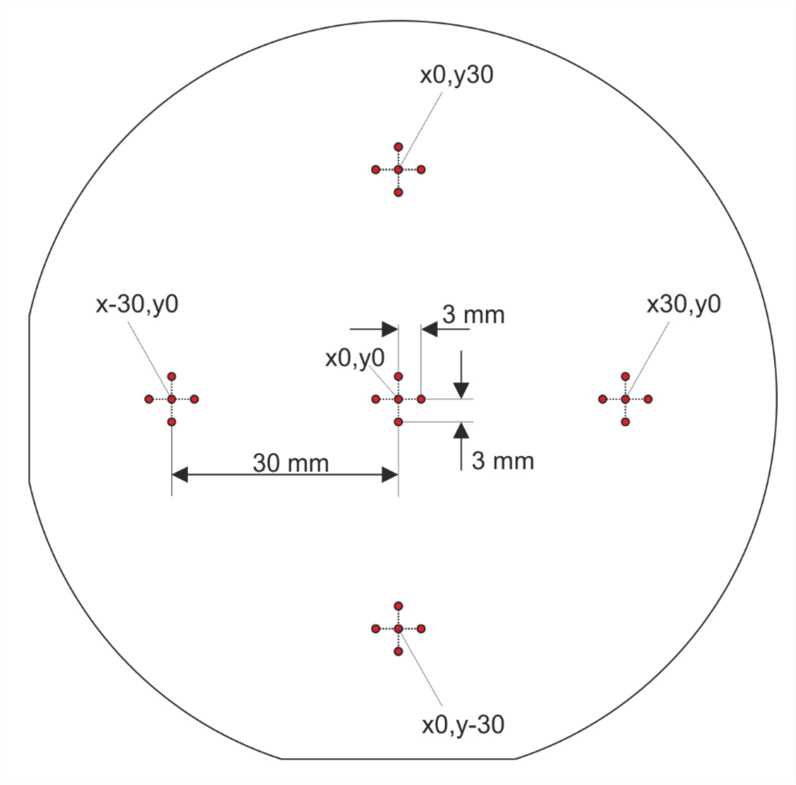

Figure S5. Schematic representation of the 25 locations on 100 mm diameter silicon where SEM images were recorded for uniformity analysis of the wedge nano-machining procedure.

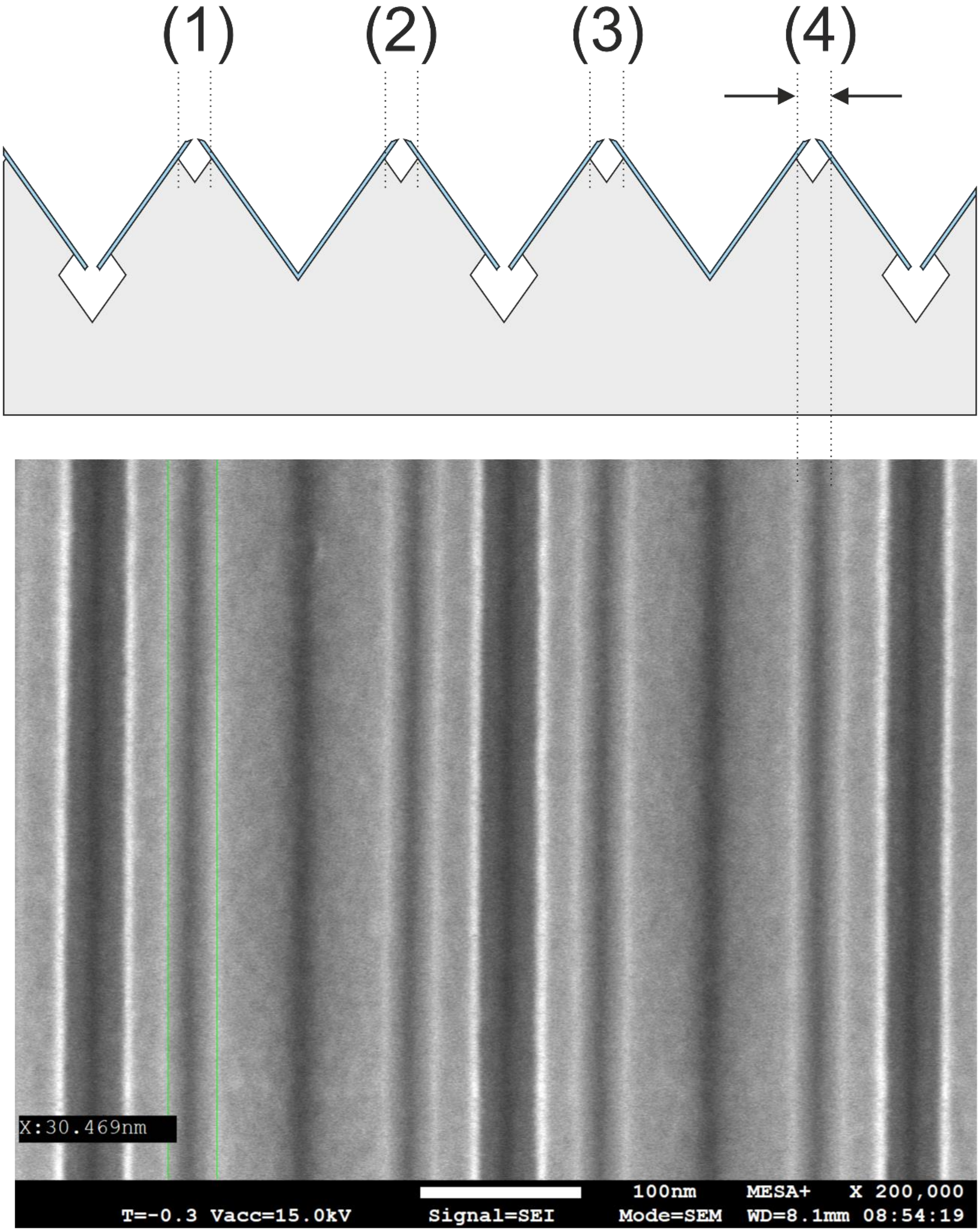

Figure S6. SEM image of wedges at location (x,y)=(0,0) (etch time in TMAH 240 s, 70 °C, 25 wt.%).

**Location**	**Width of wegdes**				**Average** (arithmetic mean)	**σ** (SD_N)
**(x,y; mm)**	**[nm]**	**[nm]**	**[nm]**	**[nm]**	**[nm]**	**[nm]**
0,0	30.5	31.4	32.0	30.8	31.6	0.4
3,0	31.9	32.0	32.0	31.7		
-3,0	31.9	31.2	31.8	31.9		
0,3	31.4	31.9	31.8	31.9		
0,-3	31.9	31.3	31.7	31.4		
30,0	28.1	30.2	29.6	30.2	29.9	0.8
33,0	31.9	30.1	29.6	29.6		
27,0	29.4	29.4	29.5	30.4		
30,3	30.5	30.2	29.6	30.1		
30,-3	29.1	29.6	31.2	30.2		
-30,0	28.1	31.2	31.7	31.2	31.1	1.1
-33,0	31.9	31.3	31.2	31.8		
-27,0	29.4	31.7	31.6	31.8		
-30,3	30.5	31.8	31.8	31.7		
-30,-3	29.1	31.8	31.2	31.8		
0,30	28.1	30.7	31.9	31.8	31.5	1.2
0,33	31.9	31.8	32.3	31.8		
0,27	29.4	32.3	32.3	32.3		
3,30	30.5	32.3	32.0	32.3		
-3,30	29.1	32.1	32.3	32.3		
0,-30	28.1	30.2	30.7	30.7	30.7	1.0
0,-33	31.9	31.3	31.8	31.7		
0,-27	29.4	31.2	30.7	31.2		
3,-30	30.5	31.2	30.7	30.7		
-3,-30	29.1	31.8	30.7	31.2		

Table S1. Widths of wedges at various locations of a substrate; the coordinates of the locations at which SEM images were taken are shown in fig. S5.

A similar procedure was followed and the TMAH etching step reduced to 60 s. On 5 locations, distributed across a substrate TEM images were recorded, see fig. S7. Based on these 5 images, the spacing between newly formed wedges was determined (after 60 s of TMAH etching). The average (i.e., arithmetic mean) spacing across a substrate is 12.0 nm with a σ-value (i.e., SD_N) of 0.5 nm.

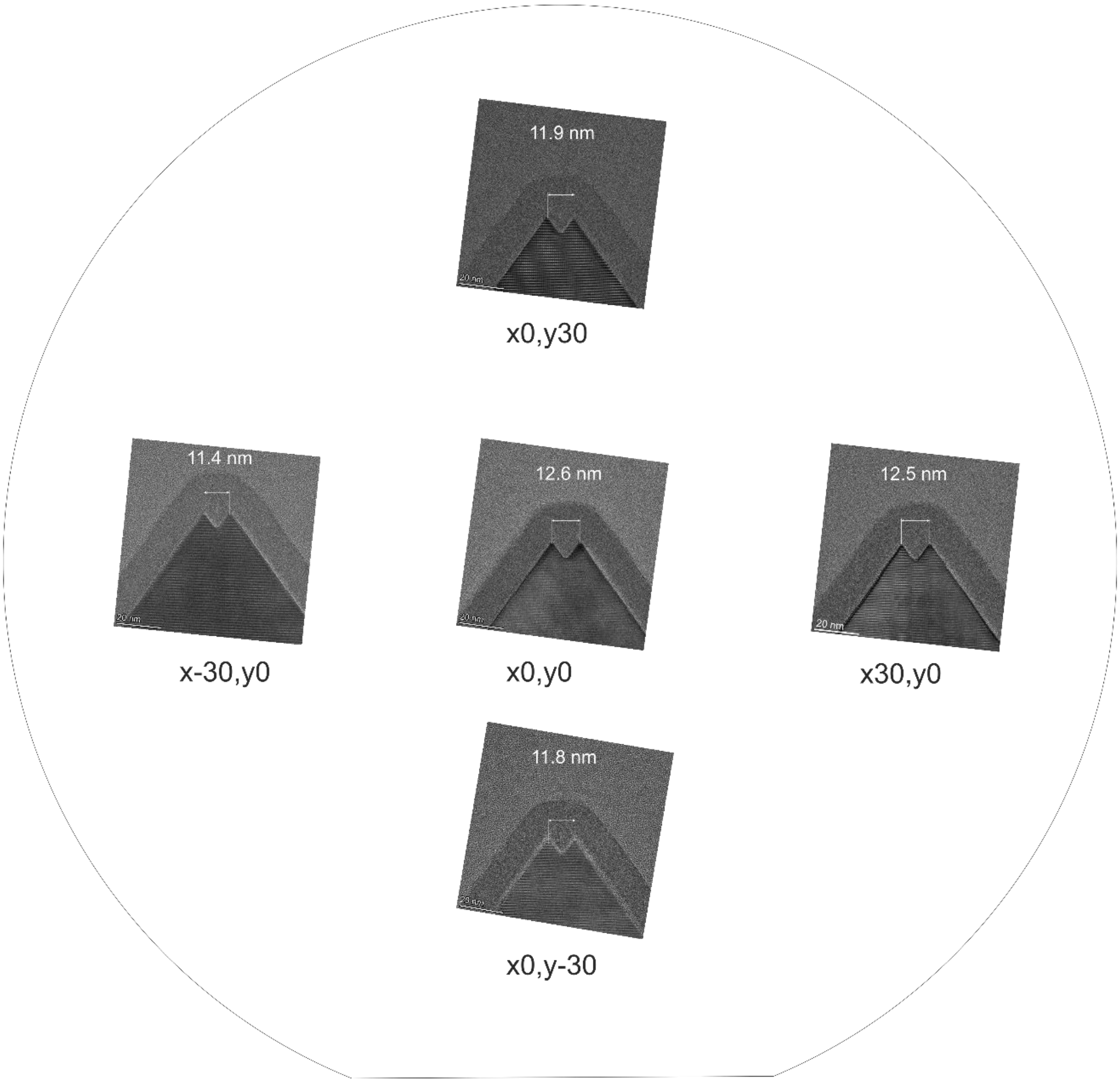

Figure S7. Schematic representation of the 5 locations on 100 mm diameter silicon where TEM images were recorded for uniformity analysis of the wedge nano-machining procedure (after etching in TMAH (70 °C, 25 wt.%) for 60 s. The (white) numbers indicate the spacing between newly formed wedges.

7. Typical integration scheme of convex corner lithography for NEMS devices fabrication

Convex corner lithography can be used for fabrication of NEMS devices. For example, high density arrays of nano-cavities can be fabricated (example taken from [S6]), as shown in figure S8. Specific details regarding the realization of nano-cavities: post to silicon nano-wedge templating, a low temperature oxidation step was done (yielding 25 nm SiO_2 at the Si(111) planes). Then a poly-silicon hard mask was deposited that was slightly oxidized and patterned using DTL, 1% HF etching and immersion in 20wt% KOH (room temperature). Subsequently, this poly-silicon mask was used to perform the silicon oxide thinning step (in 1% HF) down to 5 nm. Finally, nano-cavities were etched at the apices in TMAH through the nano-gaps created in the SiO_2 (the gap sizes measured with HR-SEM after cleaving the sample were below 20 nm).

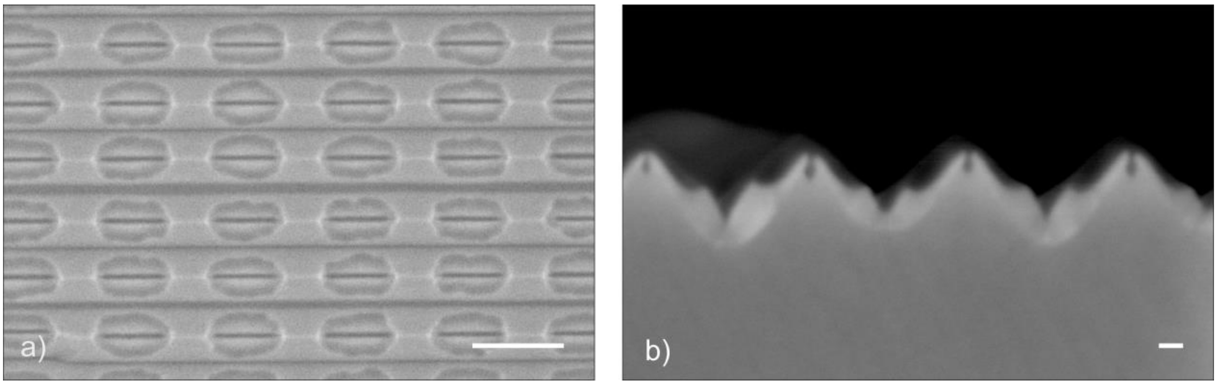

Figure S8. Examples of arrays of 5 nm thick freestanding SiO_2 flaps, that have been realized based on silicon nano-wedges templating [S1] (scale bar: a) 200 nm, b) 20 nm).

References

[S1]: Vangbo, M. ; Bäcklund, Y. Precise mask alignment to the crystallographic orientation of silicon wafers using wet anisotropic etching. *J. Micromech. Microeng.* **1996**, *6*, 279-284.

[S2]: Solak, H. H.; Dais, C; Clube, F. Displacement Talbot lithography: a new method for high-resolution pattering of large areas. *Opt. Exp.* **2011**, *19*, 10686-10691, 2011.

[S3]: Le-The, H.; Berenschot, E.; Tiggelaar, R. M.; Tas, N. R.; Van den Berg, A.; Eijkel, J. C. T. Shrinkage control of photoresist for large-area fabrication of sub-30 nm periodic nanocolumns *Adv. Mater. Technol.* **2017**, 2, No. 1600238.

[S4]: Le-The, H.; Berenschot, E.; Tiggelaar, R. M.; Tas, N. R.; Van den Berg, A.; Eijkel, J. C. T. Large-scale fabrication of highly ordered sub-20 nm noble metal nanoparticles on silica substrates without metallic adhesion layers. *Microsyst. Nanoeng.* **2018**, *4*, No. 4.

[S5]: Wilbers, J. G. E.; Berenschot, J. W.; Tiggelaar, R. M.; Dogan, T.; Sugimura, K.; Van der Wiel, W. G.; Gardeniers, J. G. E.; Tas, N. R. 3D-fabrication of tunable and high-density arrays of crystalline silicon nanostructures. *J. Micromech. Microeng.* **2018**, *28*, No. 044003.

[S6]: Berenschot, E.; Deenen, C.; Tiggelaar, R.; Gardeniers, H.; Tas, N. Waferscale fabrication of nanogaps by convex corner lithography. In *IWNA 2017, Proc. 6th Int. Workshop on Nanotechnology and Application*, Phan Thiet, Vietnam, November 8-11, 2017.
